# CO_2_ Methanation on Zeolite/Mesoporous Silica Composites Prepared from Fly Ash and Rice Husk

**DOI:** 10.3390/nano16171098

**Published:** 2026-09-01

**Authors:** Margarita Popova, Grigoria Theochari, Agnes Szegedi, Silviya Boycheva, Nikolai Marinkov, Daniela Karashanova, Daniela Kovacheva

**Affiliations:** 1 Institute of Organic Chemistry with Centre of Phytochemistry, Bulgarian Academy of Sciences, 1113 Sofia, Bulgaria; grigoria.theochari@orgchm.bas.bg; 2 National Centre of Excellence Mechatronics and Clean Technologies, 8 Bul. Kliment Ohridski, 1756 Sofia, Bulgaria; 3 Institute of Materials and Environmental Chemistry, HUN-REN Research Centre for Natural Sciences, Magyar Tudósok Krt. 2, 1117 Budapest, Hungary; szegedi.agnes@ttk.hu; 4 Department of Thermal and Nuclear Power Engineering, Technical University of Sofia, 8 Kl. Ohridsky Blvd., 1000 Sofia, Bulgaria; sboycheva@tu-sofia.bg; 5 Institute of General and Inorganic Chemistry, Bulgarian Academy of Sciences, 1113 Sofia, Bulgaria; nikiem@svr.igic.bas.bg (N.M.); didka@svr.igic.bas.bg (D.K.); 6 Institute of Optical Materials and Technologies, Bulgarian Academy of Sciences, 1113 Sofia, Bulgaria; dkarashanova@yahoo.com

**Keywords:** fly ash and waste rice husk waste valorization, composite catalysts, CO_2_ methanation, 3D-printed NiMn-catalysts

## Abstract

Composites consisting of NaX and Na-LTA zeolites and mesoporous silica in different ratios were successfully synthesized from coal fly ash and rice husk, and subsequently modified with Ni and Mn using the incipient wetness impregnation method. The initial composite and the modified materials were characterized by X-ray powder diffraction (XRD), transmission electron microscopy (TEM), energy dispersive spectroscopy (EDS), N_2_ physisorption, temperature-programmed reduction (TPR–TGA) and X-ray photoelectron spectroscopy (XPS). The formation of finely dispersed Ni, Fe spinel nanoparticles was registered in the Ni- and NiMn-containing catalysts. The presence of Mn has a favorable effect on the Ni dispersion. The support composition, including zeolite phases and the content of mesoporous silica phase, effects the formation of catalytically active metallic species for CO_2_ hydrogenation to methane. The formation of Fe^0^ and FeNi_3_ crystalline phases was detected for the reduced catalysts. Additionally, 3D printing technology was applied for the macrostructuring of the catalyst prior to the modification of the powdered supports with metal precursors, aiming to enhance their catalytic performance. The stabilization of Fe^0^ and FeNi_3_ phase dispersion in the 3D-printed samples is beneficial for long-term catalytic performance. The advantage of the 3D-printed catalyst was demonstrated, showing its higher CO_2_ consumption rate relative to the external geometric surface area compared to its powder analogue.

## 1. Introduction

Global energy demand is growing rapidly, and meeting this requirement has led to the increased combustion of fossil fuels, resulting in a significant increase in anthropogenic CO_2_ emissions. Consequently, the escalating concentration of atmospheric CO_2_ has become a worldwide concern due to its role in climate change and its contribution to the intensification of extreme weather events. Therefore, achieving carbon neutrality has attracted considerable attention due to its economic, social, and ecological implications. One optimistic approach for addressing anthropogenic CO_2_ emissions is Carbon Capture, Utilization, and Storage (CCUS), a set of technologies in which CO_2_ emitted from large-scale point sources is captured and subsequently either utilized as an industrial feedstock or stored underground [1,2]. Among CO_2_ valorization routes, the Sabatier reaction is particularly attractive, as it hydrogenates CO_2_ into methane, a readily storable energy carrier compatible with existing natural gas infrastructure [3,4]. The strongly exothermic nature of CO_2_ methanation, which favors operation at low temperatures, together with the high stability of CO_2_ chemical bonds, gives rise to energy barriers [4,5]. Consequently, highly efficient catalysts are required to fulfill a double function [6,7]: irst, to provide strong adsorption sites that effectively capture and activate CO_2_, and, second, to facilitate the dissociation of CO_2_ and H_2_ molecules, in order to promote the subsequent reaction. In this context, zeolites show strong potential for CO_2_ methanation due a range of beneficial properties, including non-toxicity, low cost, good structural stability, and uniform pore structure [8,9]. In Ni/zeolite catalysts for CO_2_ methanation, the zeolite primarily acts as a high-surface-area support that enhances metal dispersion and prevents the sintering of Ni nanoparticles. While zeolites can contribute to CO_2_ adsorption within their microporous structure, the dissociation of H_2_ predominantly occurs on metallic Ni^0^ sites. The resulting metal–support interactions therefore play an indirect but crucial role in determining catalytic activity, stability, and methane selectivity [10]. Zeolites are crystalline microporous aluminosilicates, exhibiting high thermal stability and well-defined pore structures. Due to their large surface area and tunable acidity, they are widely used as catalyst supports, where they enable the high dispersion and stabilization of active metal species. In CO_2_ methanation systems, the catalytic activity originates from the metallic phase, while the zeolite framework enhances reactant adsorption and metal dispersion, thereby improving overall catalytic performance [11,12,13]. Moreover, the use of zeolites synthesized from waste-derived materials (such as coal fly ash, slag, and industrial sludge) offers significant advantages, as they provide a cost-effective alternative to commercially available zeolites while simultaneously enhancing environmental sustainability through waste valorization [14]. For instance, coal fly ash (CFA) is a solid by-product generated from coal combustion in thermal power plants and other coal-based industrial processes. CFA is classified as non-toxic waste; however, its landfilling poses risks to soil and groundwater [15]. In recent years, the conversion of coal fly ash into zeolites has gained increasing interest because CFA-derived zeolites offer promising performance in pollutant adsorption and catalytic degradation due to their well-developed porous structure, high specific surface area, and structural stability [16,17]. Coal fly ash-derived zeolites are advantageous due to their hierarchical micro–mesoporous structure, which provides suitable sites for hosting functional particles and tuning their catalytic properties [13,18].

There has been steady progress in the synthesis of several zeolite types, such as NaA, NaP, NaX, and NaY, from CFA [19,20,21,22]. However, many CFA-derived zeolites show limited catalytic and adsorption performance toward large organic molecules. As a result, the precise control of the Si/Al ratio, often achieved through the addition of external silicon sources, is essential for tailoring zeolite properties. The adding of conventional silica additives as CFA-derived silica, silica sol, and silica aerogel can increase production costs and limit large-scale industrial implementation [23,24,25]. For this purpose, alternative silica sources can be utilized, including amorphous silica derived from agricultural waste. For example, rice husk ash (RHA) is rich in amorphous silica, which readily dissolves under elevated temperatures and atmospheric pressure, making it an effective and low-cost silicon source [26] for tailoring zeolite composition and enhancing its performance [27].

The efficiency of zeolite-based catalysts in CO_2_ methanation is significantly influenced by the incorporation of metallic promoters in addition to the active phase [9,10,28]. Numerous studies have exhibited the advantages and disadvantages of both noble and non-noble metals. Noble metals such as Rh, Ru, and Pt exhibit excellent low-temperature methanation performance, but their high cost and limited availability make them impractical for large-scale applications [3,29]. In contrast, Ni-based catalysts are cost-effective and efficient for CO_2_ methanation [30,31]. While Ni serves as the primary active metal due to its high activity, the addition of promoter metals such as cobalt (Co), iron (Fe), zirconium (Zr), yttrium (Y) and manganese (Mn) has been widely reported to enhance catalytic performance. These promoters play a crucial role in modifying both the physicochemical properties of the support and the dispersion of the active metal phase [30]. Iron (Fe) and cobalt (Co) readily alloy with nickel (Ni), forming NiFe and NiCo phases, which can enhance catalytic activity and stability, depending on the metal ratio and metal–support interactions [32]. In contrast, yttrium (Y) and zirconium (Zr) primarily act by modifying the oxide support, improving its defect structure [30]. Manganese (Mn), on the other hand, typically forms MnO_x_ species that increase surface basicity and promote CO_2_ adsorption, stands out as a simple yet effective additive for enhancing low-temperature CO_2_ methanation activity [33]. In our previous study [34], we demonstrated that the incorporation of Mn into Ni catalysts, supported on rice husk-derived mesoporous silica, significantly enhanced the catalytic activity for CO_2_ methanation. This improvement was attributed to the beneficial effect of Mn on both the surface basicity of the catalyst and the dispersion of Ni particles. These findings highlighted the critical role of the support in the formation, dispersion, and accessibility of catalytically active sites. In particular, the combination of mesoporous silica with zeolitic components can provide complementary textural and chemical properties, including enhanced CO_2_ adsorption promoted by Na^+^ charge-compensating cations within the zeolite framework [35].

Despite the promising catalytic properties of powder materials, their practical implementation is limited by difficulties associated with shaping, mechanical stability, and efficient mass transfer in fixed-bed reactors. Additive manufacturing (3D printing) provides an attractive alternative by enabling the fabrication of catalysts with precisely controlled macroscopic geometry, interconnected pore networks, and hierarchical transport pathways. Although structured 3D-printed materials have been extensively investigated in other catalytic and electrocatalytic applications, including hierarchical transition-metal phosphides and CoP-modified nanoporous carbons for the hydrogen evolution reaction [36,37], the development of 3D-printed Ni–Mn catalysts based on waste-derived zeolite/mesoporous silica composites for CO_2_ methanation remains largely unexplored.

The originality of the present work lies in the integration of waste-derived zeolitic and mesoporous silica components with a Ni–Mn bimetallic active phase within hierarchically structured, 3D-printed catalyst architectures. Compared with our previous study [34], which investigated Mn-modified Ni catalysts supported on rice husk-derived mesoporous silica and also studied their 3D printing, the present work advances this concept by introducing a hierarchical zeolite/mesoporous silica composite support derived from both industrial and agricultural wastes.

This work presents highly efficient Ni–Mn catalysts supported on zeolite/mesoporous silica composites for low-temperature CO_2_ methanation, demonstrating the synergistic interaction between CO_2_ adsorption sites associated with the hierarchical composite support and finely dispersed Ni nanoparticles promoted by Mn. Beyond the intrinsic catalytic properties, the integration of additive manufacturing enables the fabrication of structured catalysts with interconnected macroporous networks. The combination of waste valorization, hierarchical adsorption–catalytic functionality, bimetallic Ni–Fe active sites, and rational 3D structuring provides a distinctive and potentially scalable strategy for CO_2_ utilization and represents an important step toward the practical implementation of sustainable CO_2_ methanation technologies.

## 2. Experimental

### 2.1. Synthesis of the Zeolite/Mesoporous Silica Composites

In the present study, coal fly ash zeolite/mesoporous silica composites were prepared using fly ash (CFA) from lignite coal combustion and rice husks from agricultural waste. To prepare the amorphous mesoporous silica used in the synthesis of the composites, the rice husks were first washed with deionized water and then treated with 5% citric acids at 50 °C for 3 h. After drying, the rice husks were calcined at 500 °C for 6 h, with a 5 °C min^−1^ heating rate [38].

CFA used for the preparation of the composites was sampled from the electrostatic precipitators of one of the largest coal combustion power plants in R. Bulgaria, TPP AES Galabovo, with 690 MW of installed power, which has provided in recent years about 5% of the electricity consumption in the country. TPP AES Galabovo is supplied by lignite coal from Maritza East Basin with ash content on dry weight varying in the range 25–45 wt%. The chemical and phase composition of this CFA was studied previously [39]. It is a crystalline–amorphous material, with a predominant amorphous part containing approximately 74 wt% SiO_2_ + Al_2_O_3_ and about 13 wt% Fe_2_O_3_. The crystalline fraction consists mainly of quartz, mullite, anorthite, hematite, and magnetite. This CFA has been successfully used for the synthesis of NaX zeolites by applying a double-stage fusion-hydrothermal alkaline treatment [40].

In the present study, zeolite/SiO_2_ composites were prepared from CFA and rice husk-derived mesoporous SiO_2_ by the double-stage hydrothermal activation of reaction mixtures in different weight ratios of CFA/SiO_2_: 5/2 (28.6 wt% SiO_2_) and 5/3 (37.5 wt% SiO_2_), referred to hereinafter as ZS20 and ZS30, respectively. The synthesis procedure was carried out as follows. Solid mixtures of CFA and SiO_2_ were calcined, adding NaOH powder (NaOH quantity corresponds to 2.5 mol/L alkalinity during hydrothermal activation stage) at 550 °C in Ni crucibles for 1 h. The resulting solid charges were cooled, crushed and dispersed in distilled water to an alkalinity of 2.5 mol/L. As-prepared reaction slurries were homogenized by ultrasonic treatment for 25 min and were charged in hydrothermal vessels of stainless steel with inner Teflon spots. The reaction mixtures were conditioned at room temperature for 24 h and were subjected to hydrothermal activation for 6 h. The resulting powder samples were removed from the reaction mixtures by filtration, washed with distilled water until neutral, dried at 105 °C and subjected to subsequent studies. All subsequent studies were performed on average samples of the combined amounts obtained from three parallel syntheses of each reaction mixture. The possibility of Ni contamination from the crucibles during the alkaline fusion step was considered in our previous studies. In particular, the chemical composition of CFA-derived zeolites was determined by atomic emission spectroscopy in our previous work [39]. No Ni was detected in the zeolitic products, indicating that no detectable Ni contamination occurred during the alkaline fusion treatment in Ni crucibles. Additionally, the obtained composites were analyzed by XRF and no detectable Ni contamination was observed in the CFA/SiO_2_ support before Ni modification. The results are added in Supplementary Materials (Table S1).

The resulting powder composites (ZS20, ZS30) were applied as carriers for the development of powder and 3D-printed catalysts.

### 2.2. Development of Powder Catalysts by Modification of the Zeolite/Mesoporous Silica Powder Composites with Nickel and Manganese

The obtained zeolite/mesoporous silica powder composites (ZS20 and ZS30) were modified with nickel and nickel/manganese by incipient wetness impregnation. For this purpose, the solutions of nickel nitrate and manganese nitrate were prepared and mixed with the support for loading of 7 wt% Ni or 7 wt% Ni and 3 wt% Mn. The samples were left to dry and then calcined at 400 °C for 4 h in air with heat rate 3 °C min^−1^ for the elimination of the precursor salt. The obtained catalysts were indicated as Ni/ZS20, Ni/ZS30, NiMn/ZS20 and NiMn/ZS30.

### 2.3. Development of 3D Catalysts by Modification of the 3D-Printed Composite with Nickel and Manganese

The zeolite/mesoporous silica ZS20 composite was selected for 3D printing based on the appropriate combination of the microporous structure of the zeolite with the high surface area and accessible mesoporosity of the silica component, providing a hierarchical porous architecture that is advantageous for mass transport and the dispersion of active species.

#### 2.3.1. Preparation of a 3D STL Model of a Gyroid Structure

A three-dimensional (3D) STL model of a gyroid structure was designed using computer-aided design (CAD) software (Autodesk FUSION 360) for additive manufacturing. In the fabricated geometry, the composite material constituting the gyroid structure accounts for approximately 20% of the cross-sectional area, while the remaining 80% corresponds to open space. Despite this relatively low volume fraction, the gyroid architecture provides a significantly high specific surface area due to its continuous and highly interconnected porous structure. The structures were fabricated using a modified MSLA (masked stereolithography) 3D-printing system based on the Elegoo Mars 2 Pro, (Elegoo, Shenzhen, China) platform. The main specifications of the printer are as follows: build volume of 65 × 120 × 180 mm, pixel resolution of 1620 × 2560, UV light wavelength of 405 nm, and a layer thickness of 60 μm. A high-viscosity photopolymer composite resin (~20,000 cP) was used for printing. Due to its limited fluidity and the pronounced tendency for particle agglomeration, the material could not adequately flow or self-level under gravity alone. To address this limitation, a mechanical stirring mechanism was integrated into the printer. This movable stirrer operates after the fabrication of each layer, during the platform retraction phase, ensuring the continuous mixing and uniform redistribution of the composite resin across the surface.

#### 2.3.2. Composition for 3D Printing

The synthesized composite was first additionally ground using a high-speed blade mill operating at 18,000 rpm, followed by sieving through a 100-mesh screen to ensure uniform particle size distribution. The sieved powder was subsequently dried at 150 °C for 2 h to remove residual moisture.

The dried composite was then incorporated into a photopolymer resin system and homogenized using an ultrasonic processor for 30 min to achieve a uniform dispersion. The final printing formulation consisted of 50 wt% zeolite/mesoporous silica composites, 10 wt% monomer (isooctyl acrylate, CAS No. 29590-42-9), 25 wt% oligomer (urethane acrylate, CAS No. 82116-59-4), 5 wt% photoinitiator (bis(4-methoxybenzoyl)diethylgermanium, CAS No. 1207515-90-9), and 10 wt% solvent (isopropanol).

Although urethane acrylate-based photopolymer systems are relatively low-cost, their use in this study was enabled by the thin-walled geometry of the designed 3D structures. Due to the high viscosity of the composite resin and the tendency for air entrapment, the mixture was degassed in a vacuum chamber for approximately 2 h to remove trapped air bubbles.

Following fabrication via MSLA 3D printing, a so-called “green body” was obtained. The printed parts were first dried at room temperature for approximately 48 h, followed by vacuum drying for an additional 12 h to ensure complete solvent removal. Subsequently, the polymeric binder was thermally removed through controlled heat treatment in a furnace, with the temperature gradually increasing up to 550 °C.

The following formed the analysis of the hierarchical porous network of the zeolite gyroid, bridging the software-designed parameters with the experimental post-sintering data:

1. Design vs. Sintering Shrinkage: The TPMS gyroid model was designed with a 20% theoretical volume fraction (infill), leaving 80% of the volume as open macro-channels. To evaluate dimensional stability, the green body (Ø 50 mm × 20 mm) was compared to the final sintered ceramic substrate (Ø 38 mm × 15 mm). The linear sintering shrinkage was 24.0% in the XY-plane (diameter) and 25.0% along the Z-axis (height). This highly isotropic shrinkage behavior demonstrates excellent geometric fidelity and confirms that the low peeling forces of the ACF film during MSLA printing successfully prevented structural distortions or layer shifting.
2. Hierarchical Porosity and Density: A combined geometric-hydration method was used to characterize the final sintered matrix (total volume of 17.01 cm^3^). The mass difference between the dry substrate (4.14 g) and the water-saturated substrate (16.0 g) reveals that the sintered zeolite walls themselves possess an open intra-strut porosity of 69.72%, with a final bulk density of 0.243 gcm^−3^. This confirms the fabrication of a high-performance hierarchical porous catalyst substrate, where the software-defined macro-channels (80% theoretical voids) ensure low pressure drop, while the thermal removal of the photoresin binder generates an extensive secondary 69.72% open micro-/mesoporous network within the walls to maximize active catalytic sites.

#### 2.3.3. Modification of the 3D-Printed Composite with Nickel and Manganese

The obtained 3D-printed zeolite/mesoporous silica composite (ZS20) after calcination for the removal of polymeric binder was modified with nickel and nickel/manganese by incipient wetness impregnation. The amounts of Ni and Mn salts used for incipient wetness impregnation were calculated based on the mass of the calcined 3D-printed composite, which consisted only of zeolite/mesoporous silica. The nickel nitrate and manganese nitrate solutions were prepared and added dropwise onto the support to achieve Ni and Mn loadings of 7 wt% and 3 wt%, respectively. The sample was left to dry and then calcined at 400 °C for 4 h in air at the rate 3 °C min^−1^ for the elimination of the precursor salt. The catalyst was designated as 3D-NiMn/ZS20.

XRF measurements confirmed the presence of 7.6 wt% Ni and 3.2 wt% Mn contents in powder NiMn/ZS20, NiMn/ZS30and 3D-NiMnZS20 catalysts.

### 2.4. Experimental Techniques for Physico-Chemical Characterizations of Zeolite/Mesoporous Silica Composites and Catalysts

In order to investigate the structure/composition catalytic activity relation, all of the prepared catalysts were fully characterized before and after the catalysis. The structural characteristics of the different phases were investigated by X-ray powder diffraction (XRD) method. The XRD patterns were recorded at room temperature using Bruker D8 Advance diffractometer (Bruker AXS, Karlsruhe, Germany) at Bragg–Brentano geometry with CuKα radiation (λ = 1.5418 Å) and LynxEye detector. The data was collected between 10 and 90 °2θ. Phase composition was determined with EVA v4 software with the reference ICDD-PDF2 (2021) database. Topas-4.2 program was used to calculate the unit cell parameters, phase quantities, and mean crystallite sizes. For the identification of crystalline phases, ICDD card numbers were used: NaX: 00-038-0237; LTA: 00-039-0222; FeNi_3_: 01-074-5840; Fe: 00-006-0696; NiO: 00-004-0835.

The textural properties of all samples were determined from low-temperature N_2_-physisorption isotherms obtained by AUTOSORB iQ-C-MP-AG-AG (Quantachrome Instruments, Anton Paar brand, Boynton Beach, FL, USA). Before the analysis, the samples were outgassed under vacuum at 350 °C for 15 h. The specific surface area was calculated from adsorption isotherm branch at relative pressure from 0.05 to 0.21 using the Brunauer–Emmett–Teller (BET) equation [41]. The total pore volume was estimated based on the amount absorbed at a relative pressure of 0.98 and according to the Gurvich rule [42]. The pore size distribution and average pore size were evaluated from the desorption isotherm branch by the Barrett–Joyner–Halenda (BJH) method.

The redox potential of the obtained catalysts was investigated by temperature-programmed reduction–thermogravimetric analysis (TPR-TGA) performed on STA449F5 Jupiter instrument (NETZSCH Gerätebau GmbH, Netzsch, Selb, Germany). Each sample was placed in a ceramic crucible and heated at a rate of 5 °C min^−1^ in a 5 vol% H_2_ in air (100 cm^3^/min) flow up to 600 °C and a final hold-up of 1 h. Prior to the TPR experiments, the samples were treated in situ at 400 °C in air flow (10 °C min^−1^) for 1 h.

The morphology, size and shapes of the catalyst’s particles, as well as the catalytic active metal phases’ distribution into the silica support, were observed with the help of a JEOL JEM 2100 transmission electron microscope (JEOL Ltd., Tokyo, Japan) at accelerating voltage 200 kV. Each catalytic powder sample was suspended in ethanol and sonicated for 3 min. After that, a micro-quantity was dropped onto a standard copper TEM grid covered with an amorphous carbon membrane. Samples were dried in ambient conditions without any further treatment. The Crystallography Open Database (COD) with Match software (Version 3.13, Crystal Impact, Bonn, Germany) is used for phase identification. An analysis of the elemental composition of the samples was carried out, as well as mapping of the elements to visualize their distribution in the volume of the composites by means of an X-MAX N80T X-ray energy-dispersive spectrometer (XEDS, Oxford Instruments NanoAnalysis, Wiesbaden, Germany) and 11 Mp ORIUS 1000 CCD Camera (Gatan Inc., Pleasanton, CA, USA).

XPS spectra have been obtained using a SPECS Phoibos 100 X-ray photoelectron spectrometer operating in Fixed Analyzer Transmission (FAT) mode with a five-channel SPECS MCD-5 detector. An achromatic X-ray source SPECS XR50 was used with an Al/Mg X-ray tube. X-ray was set to Al Kα line at 12 kV and 200 W or Mg Kα line at identical conditions. A flood gun SPECS FG22/35 was set up to minimize surface charge-related artefacts and it was set to 1eV at 150 μA. Survey spectra were collected at pass energy 40 eV, and high-resolution spectra were collected at pass energy 15 eV and accumulated from 5 passes. The Mn 2p and Ni 2p spectra were also collected with a prolonged dwell time of 1 s, increased from the typical 0.2 s, to reach a higher noise to signal ratio. The processing was made in CasaXPS with Shirley background and built-in RSF. The binding energy in the spectra was set to 284.5 eV for C1s C–C bonds in adventitious carbon.

Hydrogen chemisorption experiments were performed using a Quantachrome Instrument AUTOSORB iQ-C-MP-AG-AG. In principle, 200 mg of the sample was loaded into a U-shaped quartz reactor and placed between quartz wool plugs. The sample was pretreated first under nitrogen flow (50 mL/min) at 300 °C for 30 min with a heating rate of 10 °C min^−1^, cooled down to 50 °C, and then reduced in situ under a flow of pure hydrogen (50 mL min^−1^) at 550 °C for 1 h with a heating rate of 10 °C min^−1^. After reduction, the sample was evacuated for 30 min at 550 °C. Afterwards, the material was cooled down to 40 °C, and evacuation took place for 30 min. The exposed active metal surface was calculated based on the hydrogen uptake. A H_2_/Ni adsorption stoichiometric factor of 1/2 was used.

### 2.5. Catalytic Activity Measurements

Prior to the catalytic experiments, all samples were reduced in situ in a hydrogen atmosphere (60 mL/min) at 550 °C for 90 min. The hydrogenation of CO_2_ was performed at atmospheric pressure using a fixed-bed flow reactor. The amount of the powdered catalysts used in the catalytic experiments was 150 mg mesh with particle size 0.25–0.50 mm. The reactants were fed in the reactor with a flow rate of 30 mL min^−1^ H_2_ and CO_2_, GHSV = 12,000 cm^3^ h^−1^ g cat^−1^, with H_2_/CO_2_ ratio 4/1, and catalytic experiments were carried out in the interval 250–400 °C. The 3D-printed catalysts were studied in a reactor working with higher flow rates (50 mL min^−1^ CO_2_ and H_2_). Volumetric flow rates (GHSV) were varied at selected temperatures to optimize the operational conditions. The on-line analysis of the reaction products was performed on NEXIS GC-2030 ATF (Shimadzu Corp., Kyoto, Japan) with a VALCO Plot VPHS-D CFS-PD3053-200 (30 m × 0.53 mm × 20.0 µm) column.

## 3. Results and Discussion

Based on X-ray powder diffraction results, the synthesized composites are consisted of mainly faujasite-structured NaX zeolite, with some Na-LTA type one as well. The wide halo between 25 and 35 °2θ is characteristic of the presence of amorphous silica. The zeolite composition is slightly different, depending on the silica content of the synthesis mixture (20% or 30%). The smaller silica content resulted in the formation of NaX around 90 wt% and, in addition, 10 wt% zeolite Na-LTA. With 30 wt% silica content, i.e., a higher amount, 20 wt% zeolite Na-LTA could be observed and the faujasite content amounted to 80 wt%. The mesoporous silica/zeolite composites originally have about 10 wt% iron, which was detected by XRF analysis (Table S1), and their color is brown. As mentioned above, the raw CFA used for composite synthesis contains a significant amount of iron, which, expressed as Fe_2_O_3_, accounts for nearly 13 wt%. Iron is present in coal fly ash in the form of its crystalline phases (magnetite, maghemite and hematite) or is included in the amorphous component of CFA, with a significant part of its spinel phases transformed into hematite during the alkaline fusion of the reaction mixtures in the synthesis of zeolites and zeolite/SiO_2_ composites. The thermal transformations of iron phases in lignite CFA have been studied [43]. Our previous studies on CFA-derived NaX zeolites have demonstrated that iron-containing species originating from the raw ash are transferred to and retained in the zeolitized material, where they can occur as finely dispersed iron oxide species, extra-framework Fe species, and, at least partly, as framework-associated Fe species [44].

**Figure 1:**
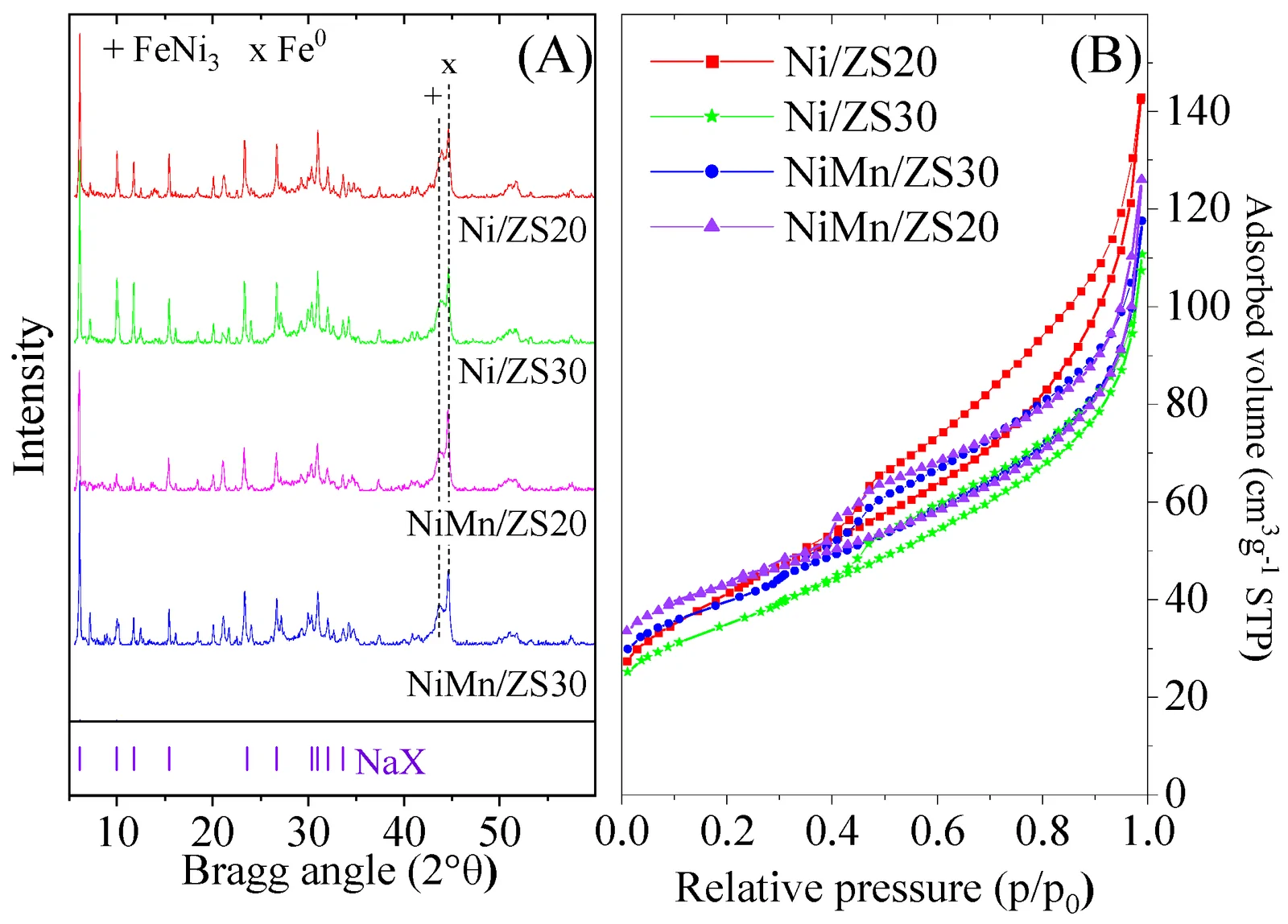
XRD patterns of reduced (**A**) and nitrogen physisorption isotherms (**B**) of the nickel- and manganese-modified zeolite/mesoporous silica composites, prepared with different silica contents.

In the composites, iron is probably in oxide form, but not in a crystalline state, since no traces of any iron oxide can be identified by XRD analysis. However, after reducing the samples in H_2_ at 550 °C, the most intensive reflection of metallic iron can be clearly observed at 44.7 °2θ (Figure 1). In the XRD patterns of modified nickel (as-prepared) samples, some crystalline NiO and nickel ferrite (NiFe_2_O_4_) can be identified with low intensity (Figures S1 and S2). The differentiation of the iron spinel phases with similar structures is difficult; therefore, high-temperature in situ XRD measurements were carried out to increase the crystallinity of the Ni/Fe phases. In the first step, the fresh samples were reduced at 700 °C in a H_2_ stream, followed by a second reoxidation step at 700 °C in air (Figures S1 and S2). By reduction at elevated temperature, the formation of Fe^0^ and a finely dispersed Fe–Ni alloy—most probably with an FeNi_3_ composition (Fm-3m (225), a_0_ = 3.575 Å)—could be observed. These observations are in line with the results of Bahgat et al. [45], who have found identical crystalline phases by the total reduction of bulk nickel ferrite in the 900–1100 °C temperature range. In our case, the zeolite/silica support helps to keep the metallic phases nanosized; therefore, the reduction to metallic state takes place at a much lower temperature. By profile-fitting the reduced samples, the crystallite size of Fe^0^ was 35 nm, whereas the Fe–Ni alloy was around 10 nm. In the XRD patterns at 700 °C, reoxidized catalysts are seen: the reflections of NiO and NiFe_2_O_4_ can be clearly identified because of their increased intensity. The presence of the ferrite phase can also be confirmed by the macroscopic magnetic behavior of the catalysts. Comparing the XRD patterns of the two catalysts, there are no differences between them, other than in the amount of zeolite A. Either way, the high-temperature treatments did not destroy the zeolite structures.

X-ray diffraction patterns of Ni/Mn-modified and H_2_-reduced catalysts are also shown in Figure 1A. The crystallinity of the zeolite phase did not change after transition metal modification, and the reflections characteristic for Fe^0^ and FeNi_3_ can also be identified. According to the XRD patterns, even the addition of manganese did not alter the dispersion or the nature of the metallic phases, and neither metallic Mn nor manganese oxides could be identified.

Nitrogen physisorption was performed in order to study the textural characteristics of the zeolite/mesoporous silica composites and their metal oxide-modified varieties (Figure 1B, Table 1). The isotherms are a combination of types II and IV, according to the IUPAC classification typical of micro–mesoporous materials [42]. Furthermore, an H3-type hysteresis loop can be observed, characteristic of the mesoporous silica constituent, having a wide pore size distribution. The parent composites have high specific surface area and micro- and total pore volume (Table 1), due to the well-organized NaX crystalline structure. In the ZS30 sample, the zeolite A content is higher; therefore, some decrease in surface area can be observed because of the narrow pore size of LTA zeolite very slowly adsorbing nitrogen.

Impregnation with Ni and Mn did not change the type of isotherms; however, this significantly decreased the micropore volume. This can be explained by the ion-exchange phenomena of sodium cations in zeolite X with nickel ions. The ionic radius of nickel is smaller than that of sodium (0.69 vs. 1.02 Å), but nickel ions tend to occupy cationic positions close to pore entrances. During reduction, a part of metallic nickel migrates to the external surface of zeolite particles, but some of the nickel nanoparticles can block the micropores of the zeolite. A partial collapse of faujasite structure due to high reduction temperature also cannot be excluded.

According to XRD investigations, nickel-modified and reduced zeolite/mesoporous silica composites contain finely dispersed nickel iron alloy phases and highly crystalline metallic iron. A significant reduction in the micropore volume of zeolites was also shown upon metal modification, probably due to pore blocking by metal nanoparticles. The addition of manganese did not have an apparent influence on textural properties.

**Figure 2:**
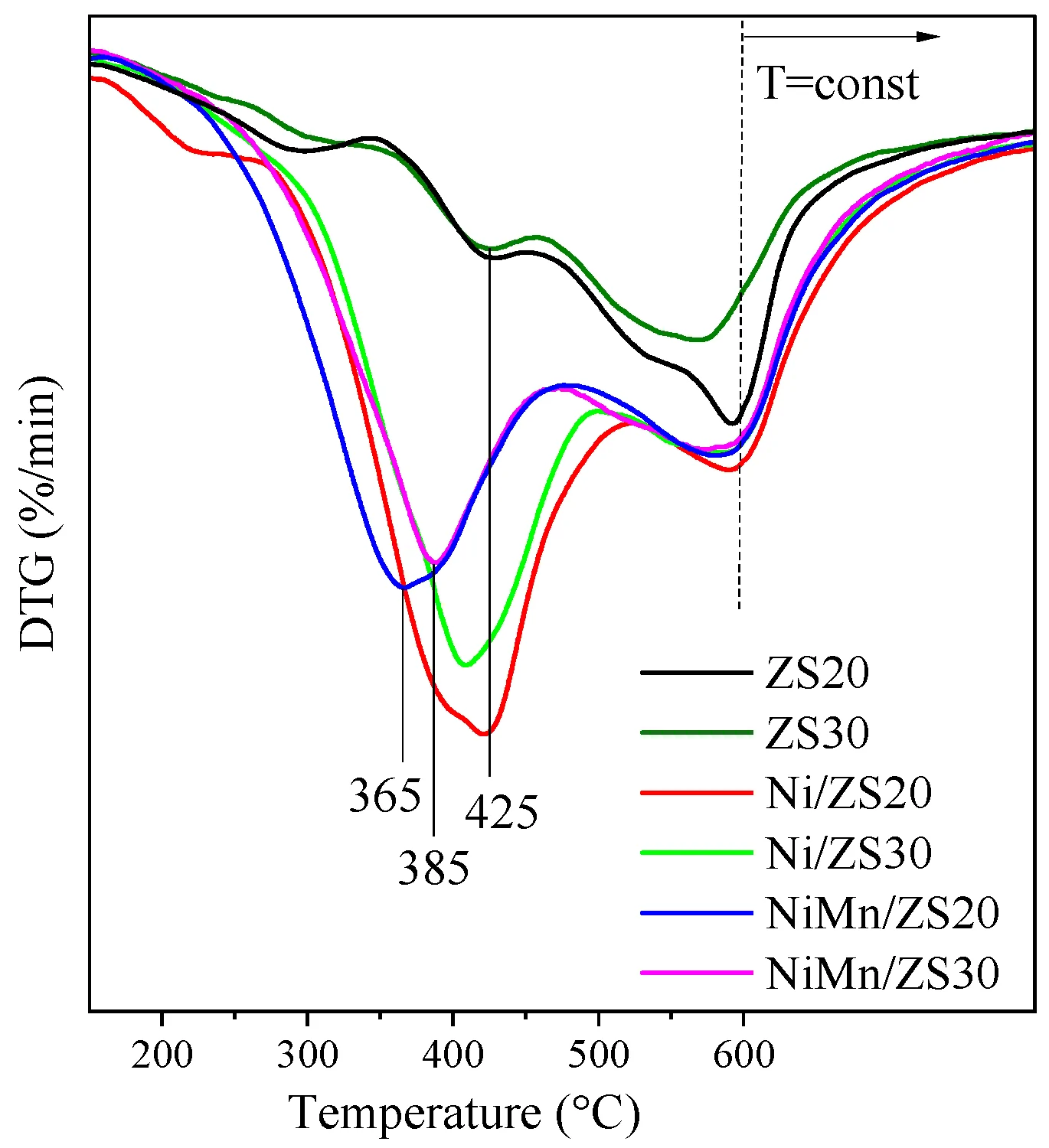
TPR profiles of the parent zeolite supports and their Ni/Mn-modified varieties.

The reducibility of metal species, metal–support interactions, and the formation of alloyed phases were examined by temperature-programmed reduction (TPR) (Figure 2). The parent zeolites show a smaller and a more intensive peak at 400 and 600 °C, respectively. The first one at 400 °C can be identified as the reduction of finely dispersed iron oxide nanoparticles on the external surface of the zeolite, and the higher-temperature one is attributed to a hardly reducible iron species, probably in the ionic position in the zeolite lattice. Iron originates from the coal fly ash used as a raw material for zeolite composite synthesis. The modification of zeolites with 7 wt% Ni resulted in the appearance of an intensive reduction peak at around 420–430 °C, and the 600 °C peak also increased. Similar to iron oxide, nickel oxide nanoparticles can be reduced between 400 and 500 °C, whereas the 600 °C peak can also be associated with the reduction of ionic nickel species in the zeolite lattice. According to the literature, separate NiO species can be reduced at the 400–500 °C interval, even above 500 °C, where they interact strongly with the support [46]. The extent of reduction was calculated by taking into account the amounts of the loaded metals and reduction of Ni^2+^ to Ni^0^, Fe^3+^ to Fe^0^ and Mn^4+^ to Mn^2+^. The addition of manganese shifted the 400 °C peak to lower values of around 30–60 °C, and the rate of reduction was decreased. The NiMn/ZS20 catalyst showed the easiest reducibility of metallic phases, with a 60 °C shift, compared to other catalysts. This indicates that the dispersion of nickel/iron nanoparticles was increased by Mn modification; the smaller nanoparticles can be reduced at a lower temperature. The second peak at 600 °C, associated with ionic species in the zeolite lattice, remained similar to other Ni zeolites. Considering the reducibility of the catalyst (Table 1), it can be observed that nickel-modified zeolite/mesoporous silica composites can be almost totally reduced to the metallic state up to 600 °C, when the reduction of iron content is considered. The addition of Mn resulted in decreased reducibility; only 81–87% of the metal content was reduced, supposing a 2e^−^ reduction of Ni to the metallic state and Mn^4+^ to Mn^2+^. According to Vrijburg et al. [47], the 2 e^−^ reduction of MnO_2_ proceeds between 300 and 500 °C in two steps, and the addition of manganese to nickel alumina catalyst shifted the reduction temperature to lower values, which is an evidence of the interaction between Ni and Mn oxidic phases. As a conclusion, they have found that the active sites in the CO_2_ methanation reaction are nickel nanoparticles decorated by finely dispersed MnO.

**Figure 3:**
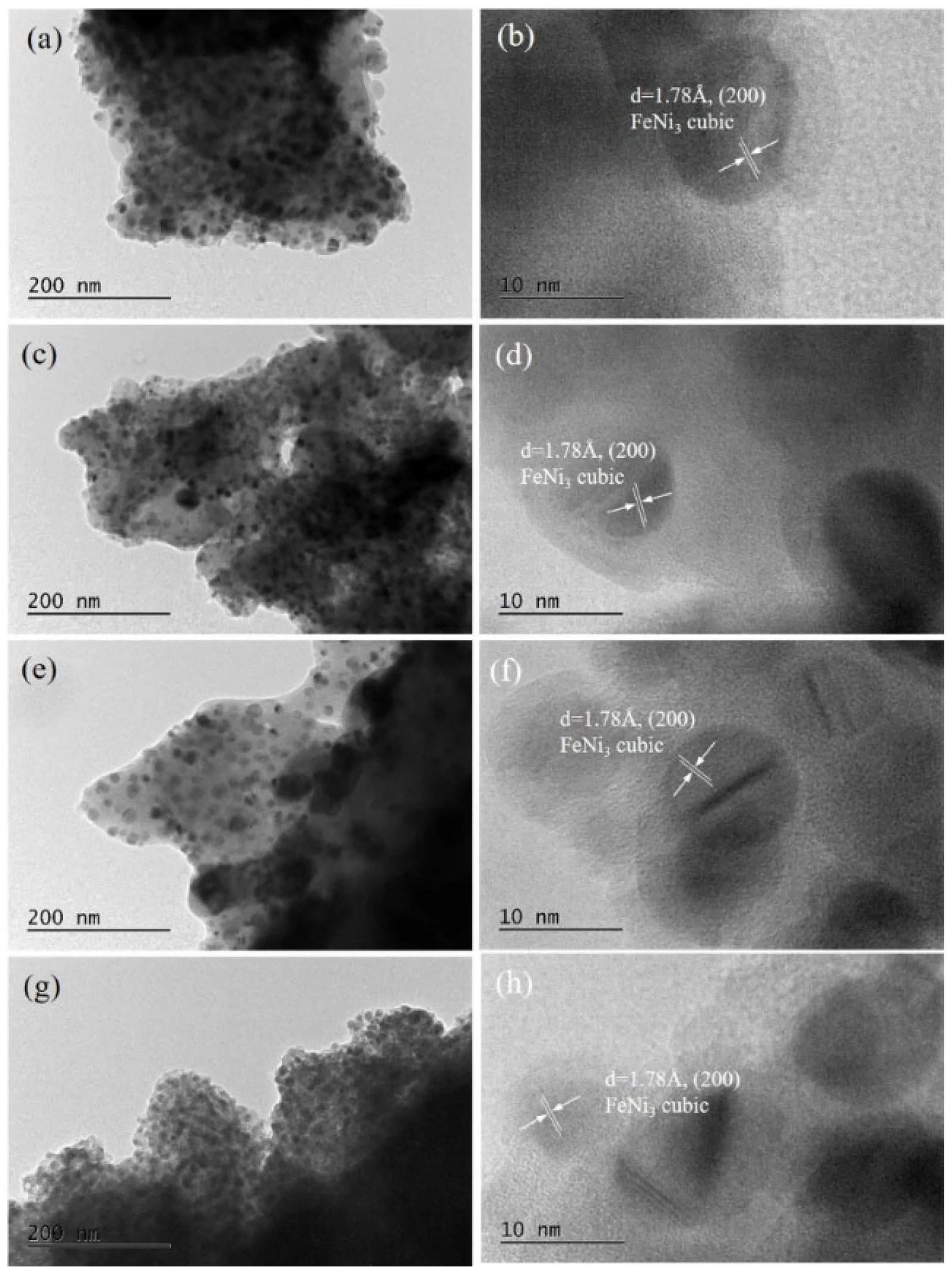
Bright-field TEM micrographs and the corresponding high-resolution TEM images of reduced Ni/ZS20 (**a**,**b**), NiMn/ZS20 (**c**,**d**), Ni/ZS30 (**e**,**f**), and NiMn/ZS30 (**g**,**h**).

The morphology of the reduced Ni and NiMn/ZS20 and ZS30 composites with SiO_2_ contents of 20 and 30%, respectively, visualized by TEM, is presented in Figure 3. Due to the difference in electronic contrast, the uniform distribution of metal nanoparticles, characterized by sizes on the order of 10 nm and less, on the particles of the zeolite and mesoporous silica supports is easily noticeable. The HRTEM images indicate the presence of the FeNi_3_ phase, constituting part of the nanoparticles, thus confirming the established data with X-ray diffraction for these samples.

**Figure 4:**
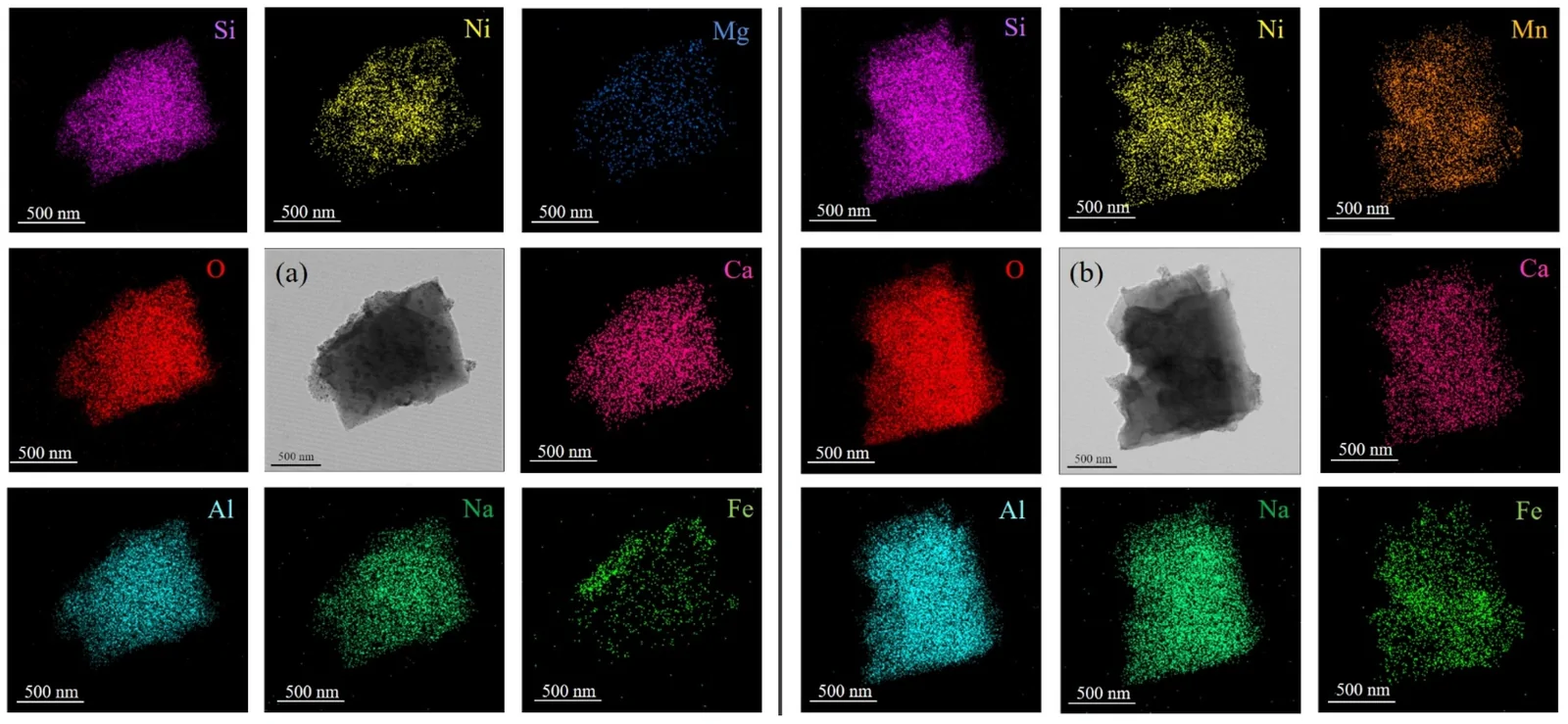
Bright-field TEM micrographs (central images) and the corresponding elemental mapping of reduced Ni/ZS30 (**a**) and NiMn/ZS30 (**b**).

For the composite with a higher mesoporous silica content of 30%, energy-dispersive spectroscopy was also performed. The distributions of elements in selected particles of Ni/ZS30 and NiMn/ZS30 composites are shown in Figure 4a and Figure 4b, respectively. The elements from the support are Si, Al, Na, Ca, Mg, O, Fe, Ni and Ni/Mn—from the deposited metals in both composites. It is seen that Ni and Fe are concentrated in the same areas of the particles, especially in the NiMn/ZS30 sample, which is a prerequisite for the formation of the mixed phase of the two metals, proven by the HRTEM and XRD.

The surface composition of the reduced Ni-modified (Ni/ZS20, Ni//ZS30) and NiMn-modified catalysts (NiMn/ZS20 and NiMn/ZS30) was studied by XPS. The spectra are presented in Figure S3, and the data are summarized in Table 2. The Ni 2p XPS spectrum demonstrates that nickel is present predominantly as Ni^2+^ incorporated into a Ni–Fe spinel structure, most likely NiFe_2_O_4_. The binding energy shift, strong satellite features, and characteristic spin–orbit splitting collectively indicate that Ni occupies octahedral coordination sites and participates in significant electronic interaction with Fe^3+^ cations through the oxygen lattice. These features confirm the formation of a well-defined spinel phase. The binding energy of the Mn 2p_3/2_ peak at ~641.6 eV (Figure S4) is generally attributed to Mn^3+^ species, although it may also indicate the coexistence of Mn^4+^, depending on the exact chemical environment. In mixed transition metal oxides, such as Ni–Fe spinel systems modified with manganese, this binding energy typically reflects a mixed valence state (Mn^3+^/Mn^4+^) rather than a single oxidation state. The Fe 2p XPS spectra of the Mn-containing samples (Figure S5) show the presence of a characteristic band at ~712 eV, which is attributed to the Fe 2p_3/2_ core level and is typical of Fe^3+^ species in oxide environments. This binding energy is in good agreement with reported values for iron in spinel structures such as NiFe_2_O_4_, where Fe predominantly exists in the trivalent oxidation state.

Considering the surface composition of the catalysts (Table 2), it was found that the surface nickel content exceeds the nominal value when it is converted to wt%, whereas manganese corresponds to the loaded one and iron shows much lower concentration on the surface compared to the bulk one. These results indicate that most of the iron is incorporated into the zeolite lattice; however, the nickel and manganese are deposited mainly on the surface, as also evidenced by TEM images.

**Figure 5:**
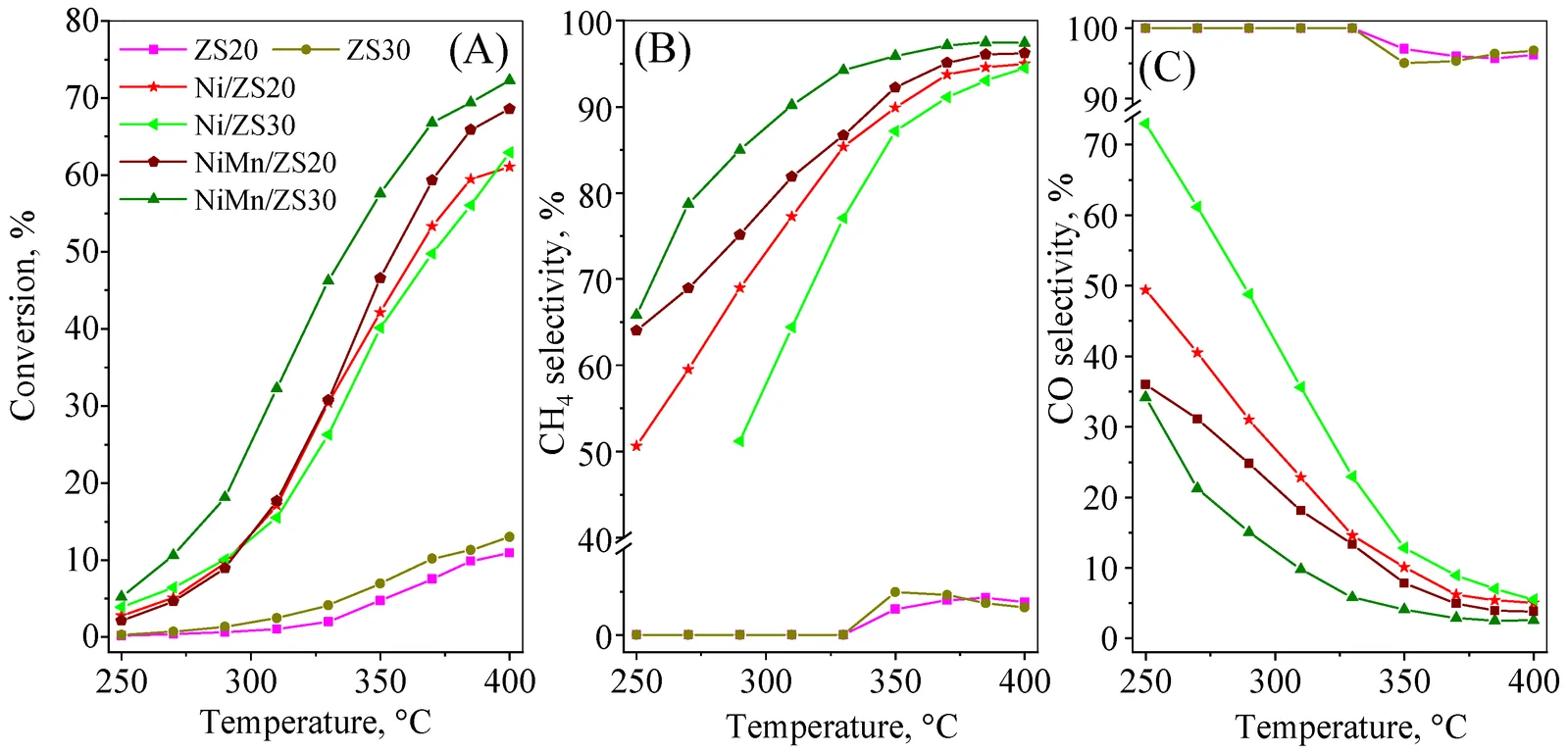
Catalytic activity (**A**), selectivity to methane (**B**), and selectivity to CO (**C**) of the zeolite/mesoporous silica composites and their transition metal-modified varieties in hydrogenation of CO_2_.

The catalytic performance of the zeolite/mesoporous silica composites and their Ni/Mn-modified analogs was systematically investigated in CO_2_ methanation. The CO_2_ conversion and the methane selectivity as a function of the temperature are presented in Figure 5. The study reveals a clear correlation between catalyst composition, reaction temperature, and CO_2_ conversion, emphasizing the role of Ni modification in enhancing the activity and stability of the catalysts. CO_2_ conversion and CH_4_ selectivity increase with reaction temperature on all catalysts. This temperature dependence aligns with the thermodynamics and kinetics of CO_2_ methanation, a highly exothermic and temperature-sensitive reaction governed by the Sabatier reaction (CO_2_ + 4H_2_ → CH_4_ + 2H_2_O, ΔH° = −165 kJ mol^−1^).

This suggests that, at lower temperatures (<300 °C), CO_2_ conversion remains relatively low due to kinetic limitations in the activation of CO_2_ and H_2_. At moderate temperatures (300–400 °C), a significant increase in catalytic activity, attributed to enhanced CO_2_ adsorption and hydrogenation over Ni active sites, occurs. Maximum CO_2_ conversion and near-complete CH_4_ selectivity can be achieved at higher temperatures (>400 °C), as the reaction proceeds through CO formation as an intermediate, which is further hydrogenated to CH_4_.

The parent zeolite catalysts with around 10 wt% iron content exhibit low catalytic activity, indicating that the highly crystalline iron contamination of the zeolites, detected by XRD, has little effect in CO_2_ conversion. At lower temperatures, incomplete hydrogenation results in the accumulation of CO as an intermediate, leading to lower CH_4_ selectivity. At higher temperatures (>300 °C), CO hydrogenation becomes more efficient, increasing CH_4_ yield while decreasing CO selectivity. Nickel-containing zeolite catalysts show similar conversion, independent of the zeolite composition in the support reaching about 60%. However, the CH_4_ selectivity is favorable on Ni/ZS20 catalysts with higher NaX content. The sodium cations within the zeolite lattice may facilitate the adsorption and activation of CO_2_ molecules by enhancing the interaction between the polar CO_2_ species and the basic sites of the zeolite framework. This interaction can promote the formation of reactive carbonate- and bicarbonate-type intermediates on the catalyst surface. Subsequently, hydrogenation is likely to proceed on the neighboring active metal phases of the composite, where dissociated hydrogen species are readily available. The proximity between the CO_2_ adsorption sites provided by the zeolite and the hydrogen activation sites associated with the metallic nanoparticles may create a synergistic effect, leading to enhanced catalytic activity and selectivity toward methane formation.

Modification with manganese improved the catalytic activity significantly: the conversion reached 70% and the selectivity increased to a higher extent, even at lower temperatures compared to Ni catalysts. The roles of manganese can be multiple. On the one hand, it can influence the dispersion of nickel; on the other hand, it can modify the basicity of the support, playing a crucial role in CO_2_ adsorption. As was found by Vrijburg et al., we can assume that the active sites in our catalysts for the CO_2_ methanation reaction are nickel nanoparticles decorated by finely dispersed MnO [47]. The best-performing NiMn/ZS30 catalyst was subjected to CO_2_ hydrogenation under time-on-stream (TOS) conditions at 400 °C (Figure S6). No catalyst deactivation was observed during the 18 h test, while high selectivity was consistently maintained throughout the reaction.

**Figure 6:**
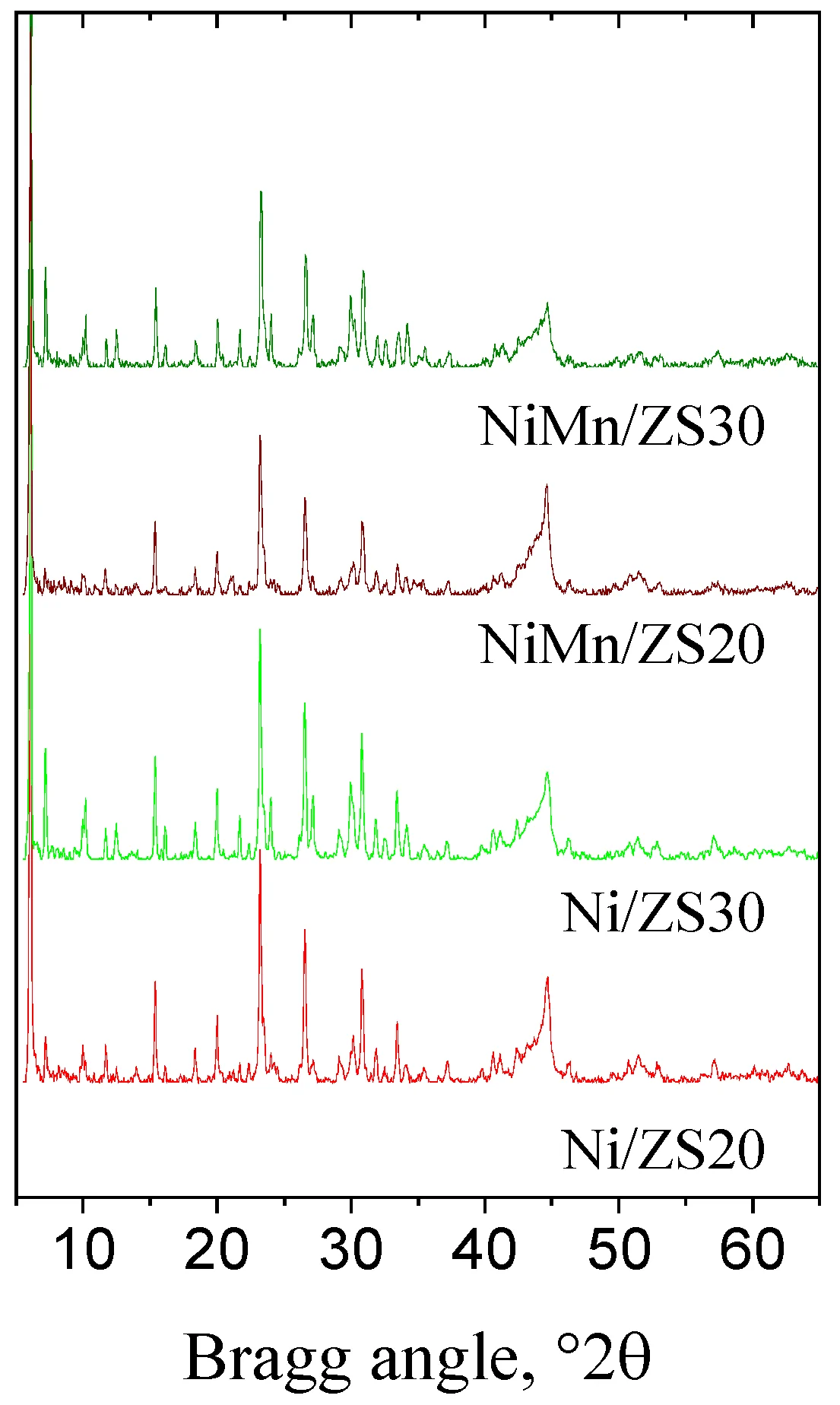
XRD patterns of the spent catalysts.

The spent catalysts were characterized by XRD and TEM methods. The XRD data for the spent catalysts are presented in Figure 6. It can be observed that the zeolite phase remained in a well-crystallized state during the catalytic run; however, there were some changes considering the nickel–iron phases. The reflection of metallic iron can be still clearly identified; however, the intensity of Ni–Fe alloy significantly decreased. This effect is probably due to the redispersion of the nanoparticles. Traces of nickel ferrite with very low intensity can be also identified due to a minor reoxidation effect of the alloy phase.

**Figure 7:**
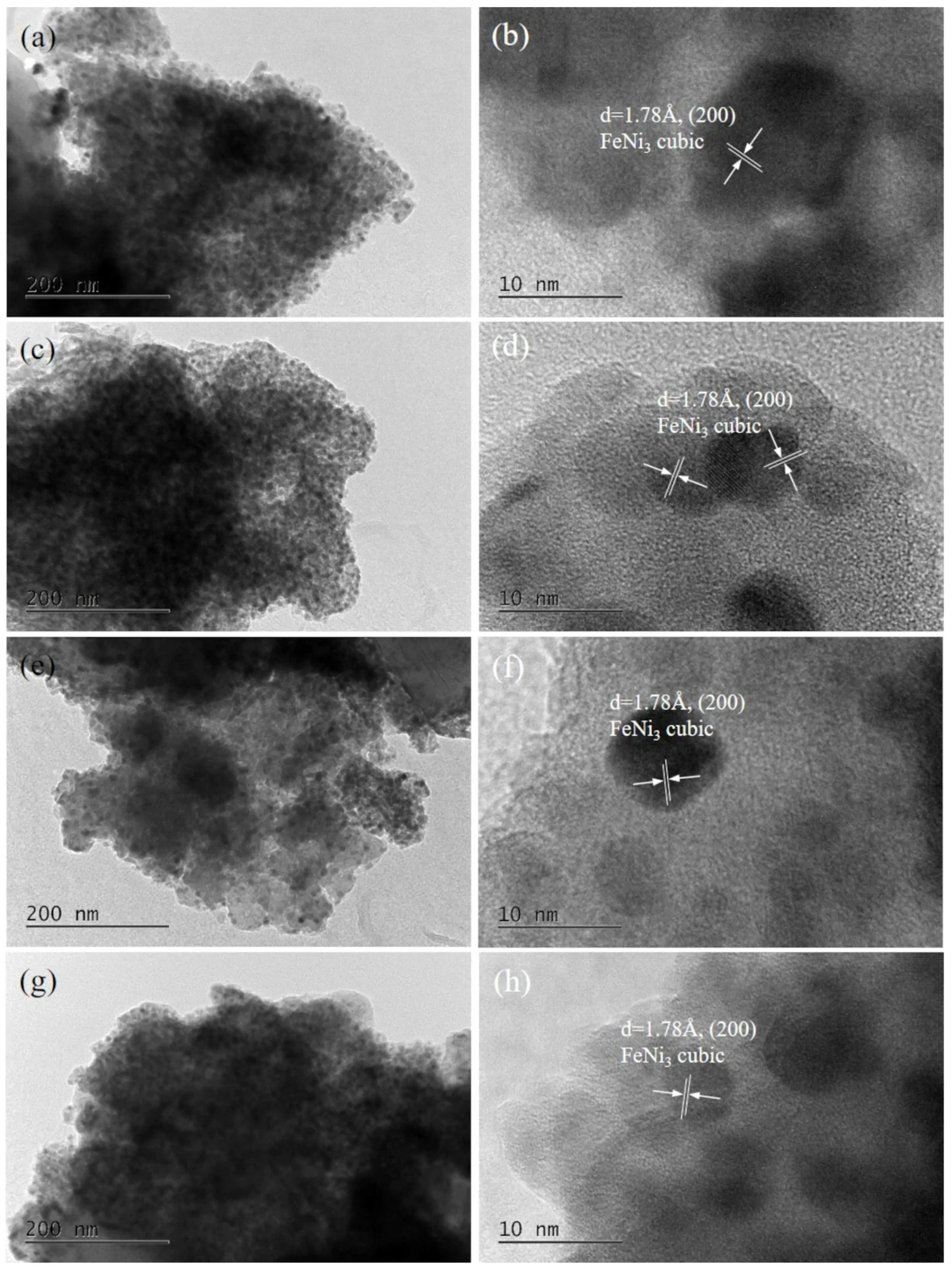
Bright-field TEM micrographs and the corresponding high-resolution TEM images of spent Ni/ZS20 (**a**,**b**), NiMn/ZS20 (**c**,**d**), Ni/ZS30 (**e**,**f**), and NiMn/ZS30 (**g**,**h**).

TEM images (Figure 7) further support this assumption, revealing the presence of smaller metallic nanoparticles on the silica surface compared to the reduced samples. The morphologies of the spent Ni/ZS30 and NiMn/ZS30 catalysts clearly demonstrate that the metal particles remained uniformly distributed over the zeolite–mesoporous silica composite support, even after the catalytic reaction.

**Figure 8:**
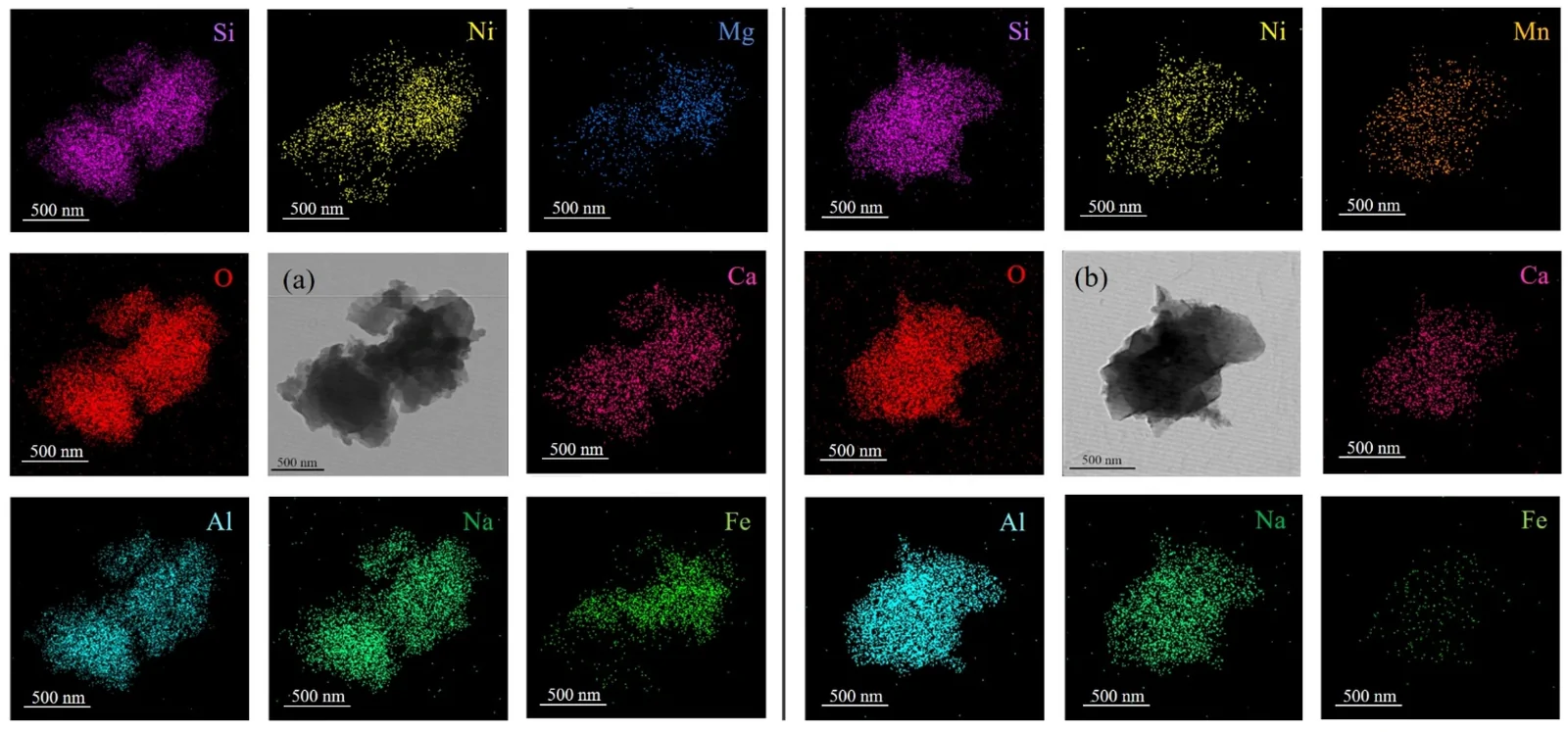
Bright-field TEM micrographs (central images) and the corresponding elemental mapping of spent Ni/ZS30 (**a**) and NiMn/ZS30 (**b**).

In addition to the morphology, the phase composition of the composite materials, preserved after their participation in the catalytic reaction, is demonstrated with high-resolution TEM. The presence of cubic FeNi_3_ and the elemental composition with the distribution of the elements in the spent catalysts are shown in Figure 8.

A 3D catalyst was prepared based on the advantageous catalytic properties of NiMn zeolite composites. The applied polymeric binder system enabled the successful fabrication of a high-quality gyroid structure, suitable for catalytic testing. The structural parameters are presented in Section 2.3.2 and Table S2. The removal of the polymeric component by heat treatment resulted in only a slight shrinkage of the body while preserving its original geometry (Table S2). Moreover, the XRF measurements confirmed the presence of 7.6 wt% Ni and 3.2 wt% Mn contents in 3D-NiMnZS20 catalysts and powder NiMn/ZS20 catalysts. The images of the 3D-printed ZS20 composite and the spent 3D-NiMn/ZS catalyst are presented in Figure S7, clearly demonstrating that the 3D-printed gyroid monolith retained its mechanical crush strength and geometric integrity after the catalytic process.

The XRD pattern of the 3D sample indicates that the crystalline structure of the NaX and Na-LTA zeolites was preserved, and the dispersion of the active metal phases (Fe^0^ and FeNi_3_) remained comparable to that of the powdered analogs, even after reduction (Figure S8). N_2_ physisorption data (Figure S9) reveal favorable modifications in the pore structure, facilitating faster and more efficient mass transport. The 3D-NiMn/ZS20 sample exhibited a specific surface area of 188 m^2^ g^−1^, of which 124 m^2^ g^−1^ corresponded to the microporous surface area.

**Figure 9:**
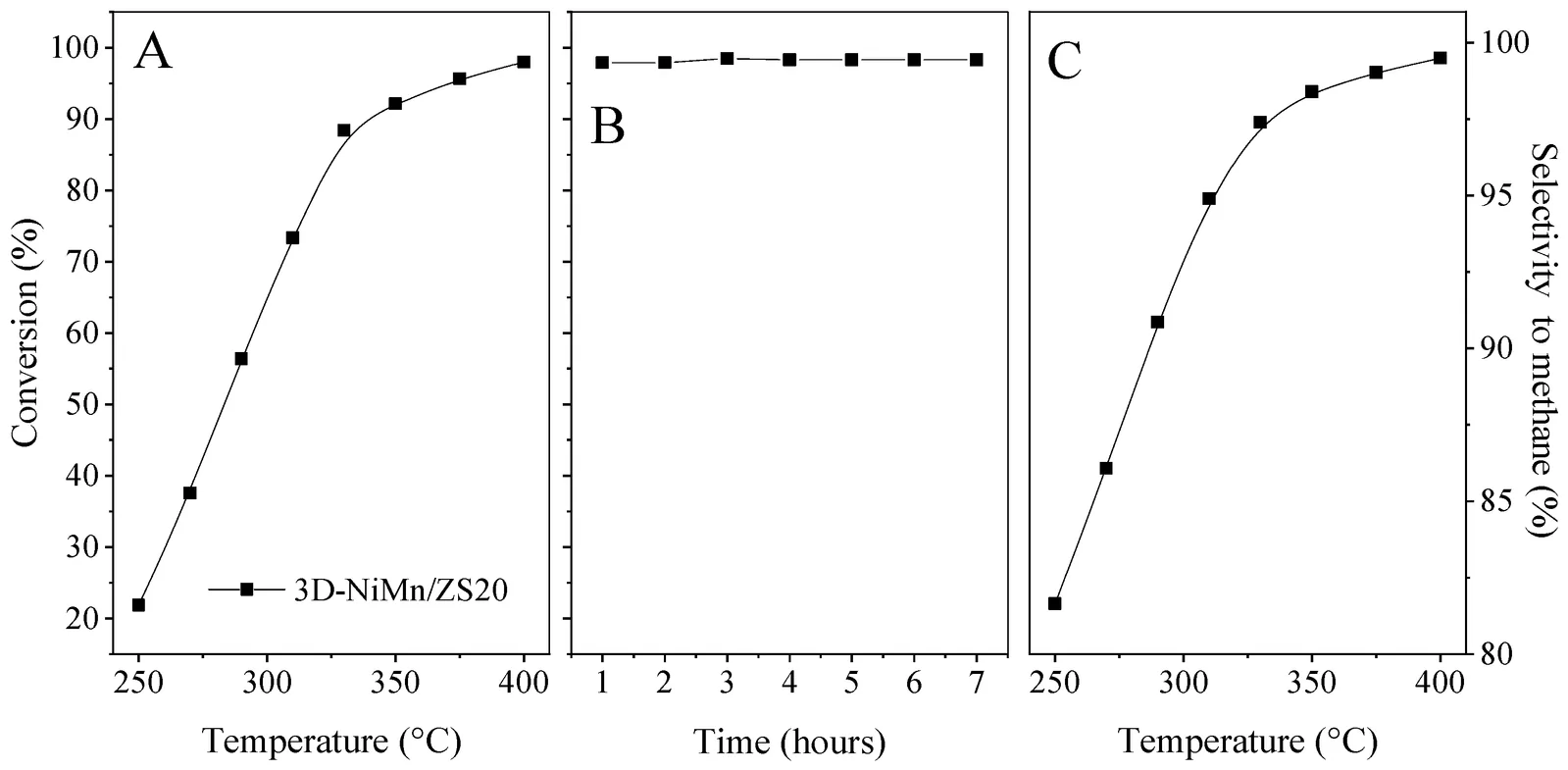
Catalytic activity (**A**), stability at 400 °C (**B**) and selectivity (**C**) of 3D-printed NiMn/ZS20 catalyst.

The catalytic testing of the 3D-printed 3D-NiMn/ZS20 catalyst (Figure 9) confirmed the beneficial effect of the applied technique, demonstrating high catalytic activity (A), selectivity (C), and stability during the time-on-stream (TOS) test (B).

The comparison with the traditional granular catalysts proved that significantly higher activity was observed for the 3D-printed NiMn/ZS20 catalyst. This catalyst had high CO_2_ conversion to methane at relatively low reaction temperature, reaching 90% conversion to methane at 320 °C, whereas the powdered NiMn/ZS20 catalyst showed 40% conversion and 95% selectivity to methane under the same temperature.

Table 3 compares the catalytic performance of various Ni-based catalysts reported for CO_2_ methanation under different reaction conditions. The evaluated catalysts exhibit significant differences in CO_2_ conversion and CH_4_ selectivity, reflecting the influence of catalyst composition, support properties, and operating conditions. Among the catalysts reported in the literature, 20Ni20Ce/MCM-41 showed one of the highest performances, achieving 85.6% CO_2_ conversion and 99.8% CH_4_ selectivity at 380 °C and a GHSV of 9000 mL h^−1^ g^−1^. Similarly, the 3D-7Ni3Mn/MS catalyst achieved 83% conversion and 99.5% selectivity at a relatively low temperature of 290 °C, indicating the beneficial effect of Mn incorporation and the 3D mesoporous structure on catalytic activity. The catalyst developed in the present work, 3D-NiMn/ZS20, demonstrated the best overall CO_2_ conversion among the catalysts compared, reaching 90% CO_2_ conversion with 96% CH_4_ selectivity at 320 °C, H_2_/CO_2_ = 4, and GHSV = 12,000 mL g^−1^ h^−1^. Notably, this performance was obtained at a lower reaction temperature than several benchmark catalysts such as Ni/γ-Al_2_O_3_ monoliths (380 °C), 15Ni/ZSM-5 (400 °C), and 20Ni20Ce/MCM-41 (380 °C). The high activity under moderate operating conditions suggests enhanced CO_2_ activation and hydrogenation over the Ni–Mn active sites supported on the hierarchical ZS20 structure. Although the 5Ni/5A catalyst exhibited complete methane selectivity (100%), its CO_2_ conversion was limited to 85%, while 10Ni/BEA showed the lowest activity, with only 33% conversion, despite maintaining relatively high methane selectivity (88%).

Compared with the structurally related 3D-7Ni3Mn/MS catalyst, the 3D-NiMn/ZS20 catalyst provided a higher CO_2_ conversion (90% versus 83%) under the same GHSV and pressure conditions, although with slightly lower methane selectivity (96% versus 99.5%).

Overall, the results demonstrate that 3D-NiMn/ZS20 is among the most competitive catalysts reported for CO_2_ methanation, combining high CO_2_ conversion, excellent methane selectivity, and operation at moderate temperatures. The superior performance can be attributed to the synergistic interaction between Ni and Mn species, the improved dispersion of active sites, and the favorable textural properties of the ZS20 support, which enhance reactant diffusion and the availability of surface-active centers.

The hydrogen chemisorption results (Table S3) demonstrate that the NiMn/ZS20 powder catalyst exhibits a H_2_ uptake of 11.31 µmol g^−1^ and a Ni dispersion of 1.90%, which are very similar to the corresponding data for the 3D-NiMn/ZS20 catalyst, showing a H_2_ uptake of 11.23 µmol g^−1^ and a Ni dispersion of 1.90%. Thus, the 3D-printed catalyst retains approximately 99.3% of the H_2_ uptake and the same Ni dispersion as the parent powder catalyst, indicating that the printing and subsequent thermal treatment do not cause a loss of accessible Ni sites or metal particle sintering. The metal surface areas calculated per gram of total catalyst are 0.88, 0.93, and 0.88 m^2^ g^−1^ for NiMn/ZS20, NiMn/ZS30, and 3D-NiMn/ZS20, respectively. When normalized to the mass of the metal phase, the corresponding values increase to 12.64, 13.25, and 12.55 m^2^ g metal^−1^. The close agreement between the metal-specific surface areas of NiMn/ZS20 and 3D-NiMn/ZS20 further confirms that the accessible Ni surface is preserved after 3D printing.

The normalization of the CO_2_ consumption rate to the external geometric surface area provides additional insight into the performance of the powder and 3D-printed catalysts. The NiMn/ZS20 and NiMn/ZS30 powder catalysts exhibited surface-normalized CO_2_ consumption rates of 2.11 and 1.42 µmol m^−2^ s^−1^, respectively, whereas the 3D-NiMn/ZS20 catalyst showed a higher value of 2.53 µmol m^−2^ s^−1^. Thus, the 3D-printed catalyst exhibited approximately 20% higher CO_2_ consumption per unit external geometric surface area than the corresponding NiMn/ZS20 powder.

These results demonstrate that the 3D-printed architecture not only facilitates the practical application of the catalysts but also enhances mass transfer and improves the accessibility of catalytically active sites.

## 4. Conclusions

Zeolite/mesoporous silica composites containing NaX, Na-LTA, and mesoporous silica in different ratios were successfully prepared from coal fly ash and rice husk. The composites were modified with 7 wt% Ni and 3 wt% Mn using the incipient wetness impregnation method. The formation of finely dispersed nickel oxide and nickel ferrite phases was detected by XRD, while XPS analysis further confirmed the presence of the latter phase. Reduction at 550 °C led to the formation of metallic iron and FeNi_3_ alloy nanoparticles; however, the incorporation of iron and nickel cations into the zeolite structure cannot be excluded. TEM and EDS investigations confirmed the formation of silica–zeolite composites and revealed the presence of finely dispersed metallic nanoparticles homogeneously distributed on the composite surface. The sodium cations within the zeolite lattice may facilitate the adsorption and activation of CO_2_ molecules, while hydrogenation likely proceeds on the neighboring active metal phases of the composite.

The catalysts exhibited high activity, selectivity, and stability in the hydrogenation of CO_2_ to methane. The best-performing catalysts were those containing manganese, suggesting that manganese oxide particles play an important role in stabilizing the highly dispersed nickel species. The investigation of the spent catalysts indicated a partial redispersion of the FeNi_3_ alloy phase during the catalytic run, resulting in even more finely dispersed metallic nanoparticles, which contributed to the high catalytic activity and stability during CO_2_ methanation.

A successful 3D-printing approach was also applied to shape one of the most active NiMn catalysts. The advantage of the 3D-printed catalyst was demonstrated by its significantly higher activity in CO_2_ hydrogenation compared to its powdered analog. The 3D-NiMn/ZS20 catalyst achieved 90% CO_2_ conversion to methane at 320 °C, whereas the powdered NiMn/ZS20 catalyst reached only 40% conversion with 95% methane selectivity under the same conditions. The normalization of the CO_2_ consumption rate to the external geometric surface area further confirmed the better performance of the 3D-printed catalyst. Compared with the NiMn/ZS20 powder, 3D-NiMn/ZS20 exhibited an approximately 20% higher CO_2_ consumption rate per unit external surface area, demonstrating that its interconnected macroporous architecture facilitates efficient gas–solid contact, reactant transport, and the accessibility of catalytically active sites. Overall, these results highlight the potential of 3D printing to combine high catalytic activity with a structured architecture that is advantageous for practical catalyst implementation.

## Figures and Tables

**Table 1:** Textural properties and reducibility of the parent zeolite/mesoporous silica composite catalysts and their Ni/Mn-modified varieties.

Samples	S_BET_ m^2^ g^−1^	V_micro_ cm^3^ g^−1^	S_meso_ m^2^ g^−1^	TPV ^a^ cm^3^ g^−1^	Reducibility ^b^ %
ZS20	500	0.15	140	0.40	95
ZS30	470	0.13	135	0.35	97
Ni/ZS20	150	0.010	126	0.22	95
NiMn/ZS20	155	0.003	81	0.16	94
Ni/ZS30	111	0.010	108	0.16	86
NiMn/ZS30	141	0.011	115	0.18	81

^a^ Total pore volume; ^b^ assuming 2e^−^ reduction of Ni^2+^ and Mn^4+^, and 3e^−^ of Fe^3+^.

**Table 2:** Chemical composition of the catalysts based on the XPS analysis, at%.

Samples	Ni	Mn	Fe	Si	Al	Mg	Na	Ca	O
Ni/ZS20	7.7	-	1.4	14.5	12.7	0.9	2.3	2.0	58.5
Ni/ZS30	9.1	-	1.5	14.1	8.3	0.6	1.9	1.3	63.2
NiMn/ZS20	6.7	1.7	1.6	13.5	11.4	2.0	2.4	2.3	58.4
NiMn/ZS30	7.3	1.5	1.3	11.8	11.9	1.6	2.3	1.6	60.7

**Table 3:** Comparison of the catalytic performance of Ni-supported catalysts in CO_2_ methanation reported in the literature.

Catalysts	Reaction Conditions	Catalytic Properties	References
Ni/γ-Al_2_O_3_ monoliths	T = 380 °C, H_2_/CO_2_ = 4; P = 0.1 MPa; GHSV = 3462 mL g cat^−1^ h^−1^	X = 60%; S_CH4_ = 99%	[48]
5Ni/5A	T = 300 °C; H_2_/CO_2_ = 4.05 GHSV = 92 mL h^−1^ g^−1^	X = 85%; S_CH4_ = 100%	[49]
10Ni/BEA	T = 350 °C; H_2_/CO_2_ = 4 GHSV = 10,000 mL h^−1^ g^−1^	X = 33%; S_CH4_ = 88%	[50]
10Ni/ZSM-5	T = 290 °C; H_2_/CO_2_ = 4 GHSV = 2400 mL h^−1^ g^−1^	X = 76%; S_CH4_ = 75%	[51]
15Ni/ZSM-5	T = 400 °C; H_2_/CO_2_ = 4 GHSV = 43,000 mL h^−1^ g^−1^	X = 65%; S_CH4_ = 95%	[52]
20Ni20Ce/MCM-41	T = 380 °C; H_2_/CO_2_ = 4/1; P = 0.1 MPa; GHSV = 9000 mL h^−1^ g^−1^	X = 85.6%; S_CH4_ = 99.8%	[53]
3D-7Ni3Mn/MS	T = 290 °C; H_2_/CO_2_ = 4; P = 0.1 MPa GHSV = 12,000 mL g cat^−1^ h^−1^	X = 83%; S_CH4_ = 99.5%	[34]
3D-NiMn/ZS20	T = 320 °C; H_2_/CO_2_ = 4; P = 0.1 MPa GHSV = 12,000 mL g cat^−1^ h^−1^	X = 90%; S_CH4_ = 96%	Current work

## Data Availability

The original contributions presented in this study are included in the article/Supplementary Materials. Further inquiries can be directed to the corresponding author.

## Supplementary Materials

The following supporting information can be downloaded at: https://www.mdpi.com/article/10.3390/nano16171098/s1. Figure S1: XRD patterns of ZS20 (a) and as prepared NiZS20 catalyst (b), reduced at 700 °C (c), and reoxidized at 700 °C (d). Identified crystalline phases: x–purple line: NiFe_2_O_4_; +-blue line NiO; *–red line: FeNi_3_; o-green line: Fe^0^. Figure S2: XRD patterns of ZS30 (a) and as prepared NiZS30 catalyst (b), reduced at 700 °C (c), and reoxidized at 700 °C (d). Identified crystalline phases: x–purple line: NiFe_2_O_4_; +-blue line NiO; *–red line: FeNi_3_; o-green line: Fe^0^. Figure S3: Ni 2p XPS spectra of the Ni- and NiMn zeolite/mesoporous silica composites. Figure S4: Mn 2p XPS spectra of the Ni- and NiMn zeolite/mesoporous silica composites. Figure S5: Fe 2p XPS spectra of the Ni- and NiMn zeolite/mesoporous silica composites. Figure S6: Catalytic activity with TOS of the NiMn/ZS30 in hydrogenation of CO_2_. Figure S7: 3D printed ZS20 composite and 3D-NiMn/ZS20 after catalytic experiment. Figure S8: XRD of 3D-NiMn/ZS20 catalyst. Figure S9: Nitrogen physisorption isotherms of 3D-NiMn/ZS20. Table S1: Structural and dimensional properties of the zeolite gyroid catalyst substrate. Table S2: Chemical composition of the initial composites based on XRF analysis. Table S3: Results from hydrogen chemisorption for the powder and 3D printed catalysts.
