# Supplementary material for: CO2 Methanation on Zeolite/Mesoporous Silica Composites Prepared from Fly Ash and Rice Husk
Source: Nanomaterials (Basel). 2026 Sep 1;16(17):1098. doi: 10.3390/nano16171098 (PMC13566967; doi:10.3390/nano16171098)
Supplement: Supplementary file 1 [file nanomaterials-16-01098-s001.zip › nanomaterials-4489842-supplementary.pdf]

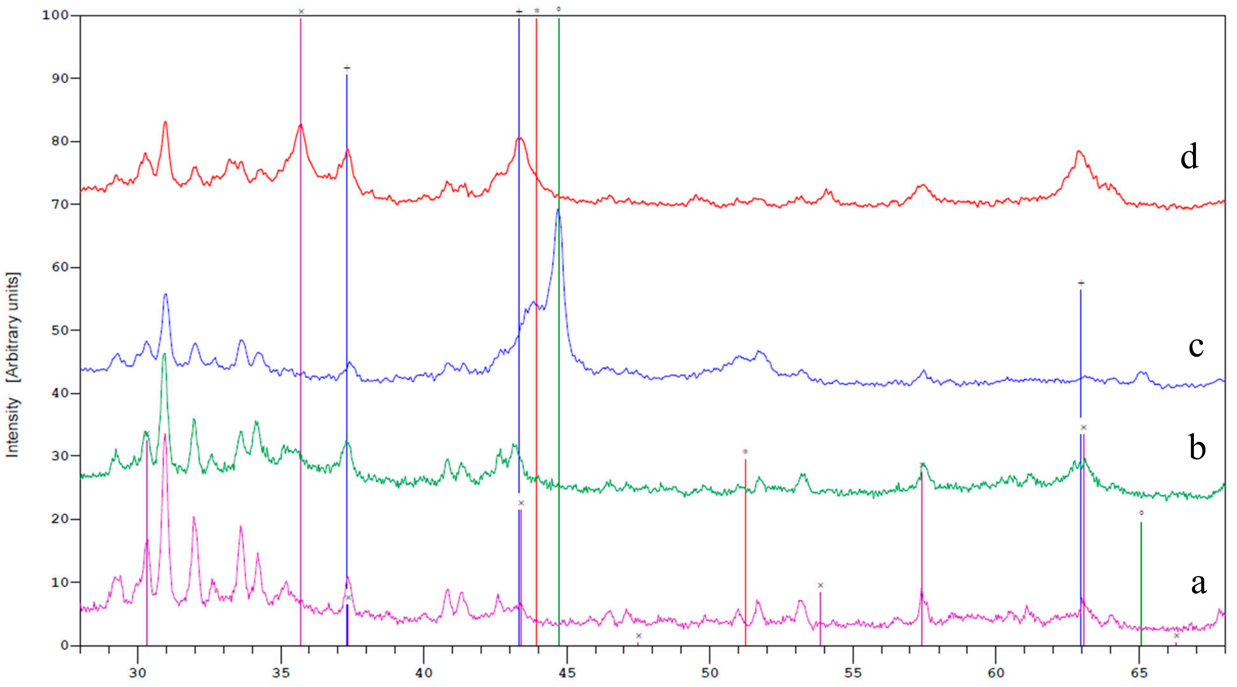

Bragg angle, °2θ

Figure S1. XRD patterns of ZS20 (a) and as prepared NiZS20 catalyst (b), reduced at 700 °C (c), and reoxidized at 700 °C (d). Identified crystalline phases: x–purple line: $NiFe_2O_4$; +-blue line NiO; *–red line: Ni_3Fe; o-green line: Fe^0.

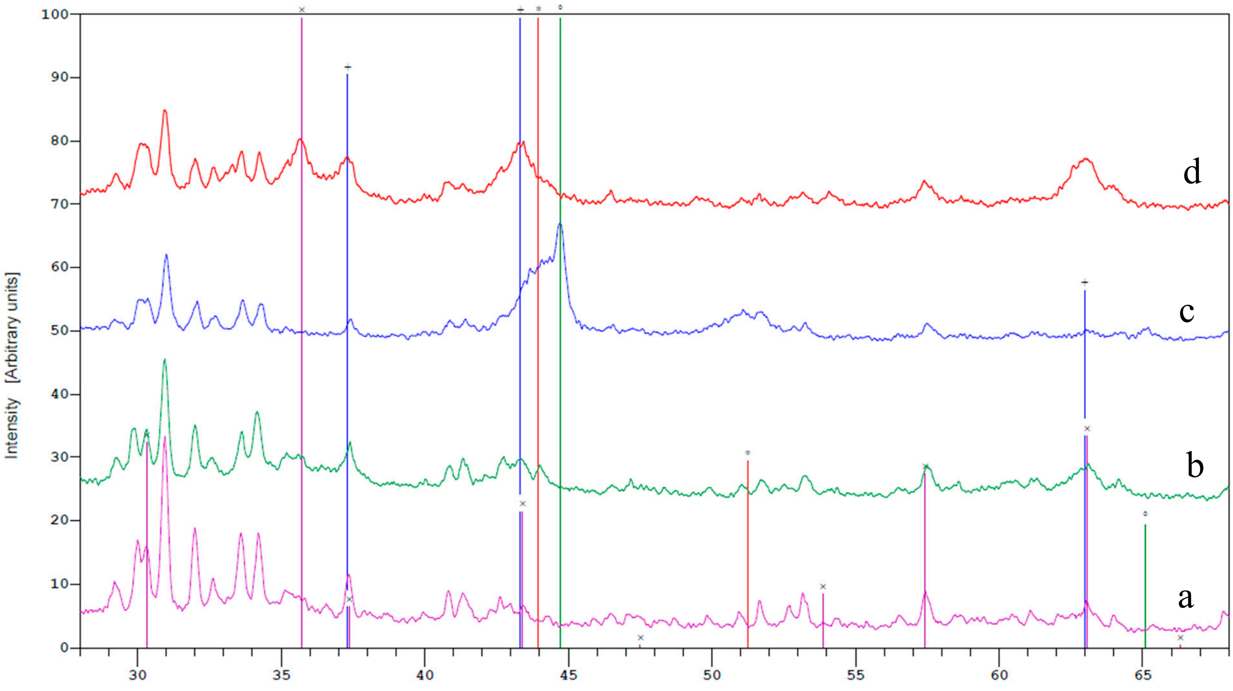

Bragg angle, °2θ

Figure S2. XRD patterns of ZS30 (a) and as prepared NiZS30 catalyst (b), reduced at 700 °C (c), and reoxidized at 700 °C (d). Identified crystalline phases: x–purple line: $NiFe_2O_4$; +-blue line NiO; *–red line: Ni_3Fe; o-green line: Fe^0.

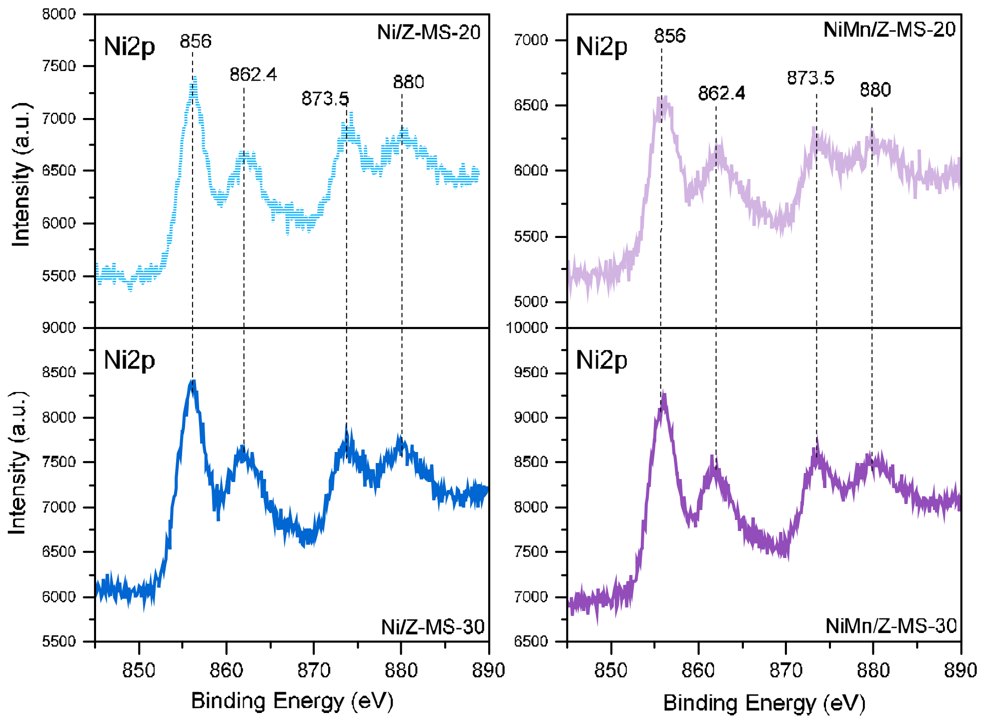

Figure S3. Ni 2p XPS spectra of the Ni- and NiMn zeolite/mesoporous silica composites.

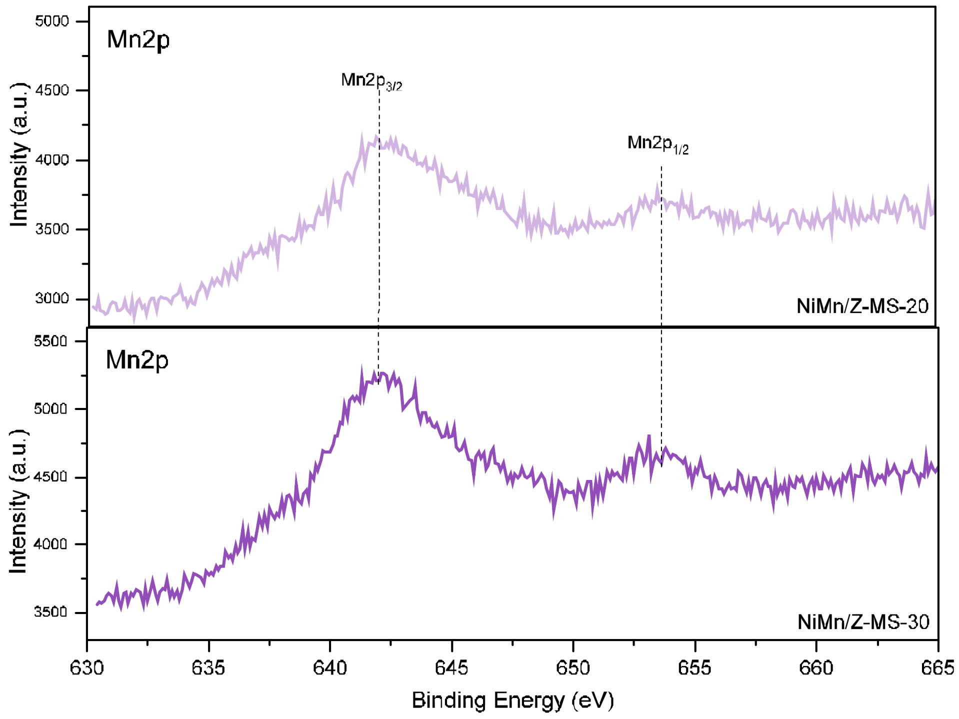

Figure S4. Mn 2p XPS spectra of the Ni- and NiMn zeolite/mesoporous silica composites.

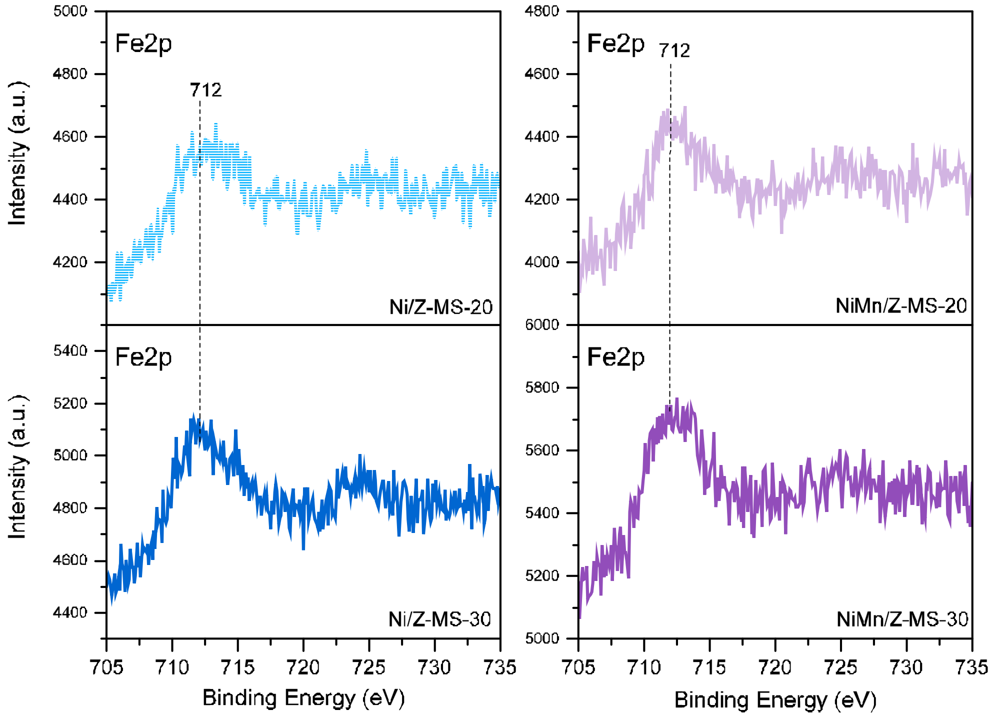

Figure S5. Fe 2p XPS spectra of the Ni- and NiMn zeolite/mesoporous silica composites.

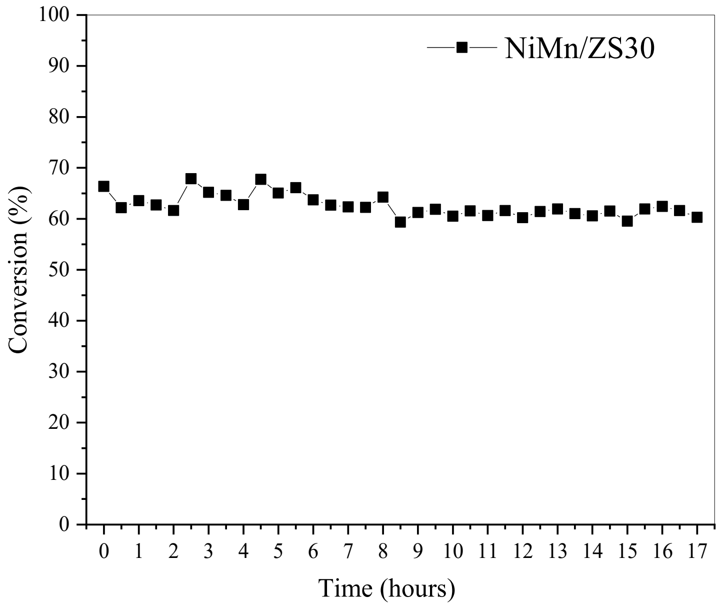

Figure S6. Catalytic activity with TOS of the NiMn/ZS30 in hydrogenation of CO_2.

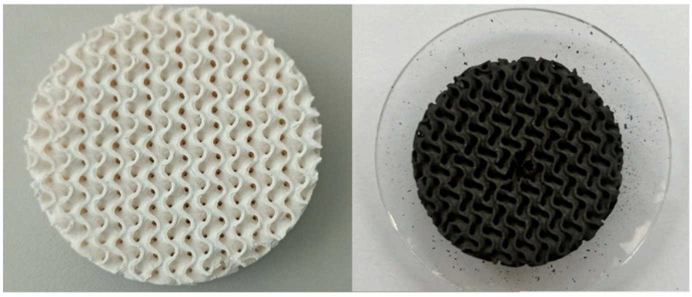

Figure S7. 3D printed ZS20 composite and 3D-NiMn/ZS20 after catalytic experiment.

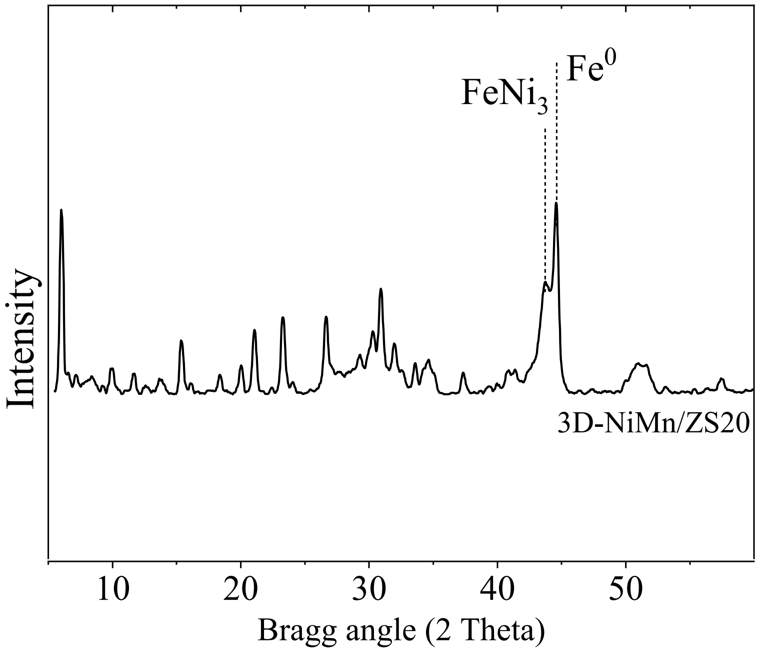

Figure S8. XRD of 3D-NiMn/ZS20 catalyst.

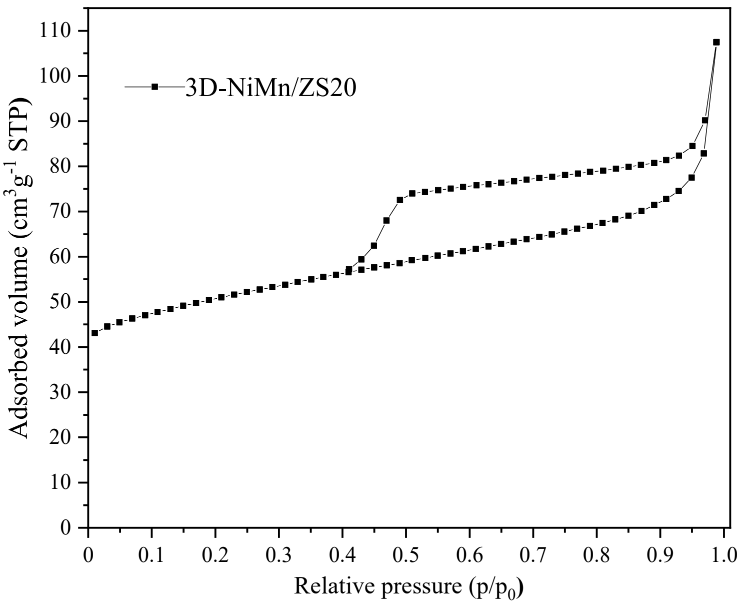

Figure S9. Nitrogen physisorption isotherms of 3D-NiMn/ZS20.

Table S1. Structural and dimensional properties of the zeolite gyroid catalyst substrate.

Property Type	Parameter	Value
**CAD Design Parameters**	Theoretical Volume Fraction (Infill)	20.0 %
	Designed Macro-void Volume	80.0 %
**Green Body Dimensions**	Diameter (Ø)	50.0 mm
	Height (H)	20.0 mm
	Layer Thickness	0.06 mm (60 µm)
**Sintered Ceramic Properties**	Sintered Diameter (Ø)	38.0 mm
	Sintered Height (H)	15.0 mm
	Linear Shrinkage (XY-plane)	24.0 %
	Linear Shrinkage (Z-axis)	25.0 %
	Sintered Total Volume (V_{total})	17.01 cm³
	Dry Mass (M_{dry})	4.14 g
	Water-Saturated Mass (M_{sat})	16.0 g
	Experimental (Ɛ)	69.72 %
	Bulk Density (p_{bulk})	0.243 g/cm³

Table S2. Chemical composition of the initial composites based on XRF analysis

Samples	Ni, wt%	Al, wt%	Si, wt%	Fe, wt%	Na, wt%	Mg, wt%	Ca, wt%
ZS20	-	9.1	19.0	10.2	8.6	0.7	4.6
ZS30	-	8.6	21.0	10.1	7.2	0.7	4.5

Table S3. Results from hydrogen chemisorption for the powder and 3D printed catalysts

Catalysts	Dispersion, %	Hydrogen uptake, µmol/g	Metal surface area*, m^2/g	Metal surface area**, m^2/g
NiMn/ZS20	1.90	11.31	0.88	12.64
NiMn/ZS30	1.98	11.85	0.93	13.25
3D-NiMn/ZS20	1.90	11.23	0.88	12.55

* calculated per gram sample; ** calculated per gram metal.
